# Supplementary material for: Negative consequences of failing to communicate uncertainties during a pandemic: an online randomised controlled trial on COVID-19 vaccines
Source: BMJ Open. 2022 Sep 7;12(9):e051352. doi: 10.1136/bmjopen-2021-051352 (PMC9453426; doi:10.1136/bmjopen-2021-051352)
Supplement: Supplementary data [file bmjopen-2021-051352supp001.pdf]

Appendix: Further analyses on outcome variables

We investigated whether announcement certainty, demographics (age, gender, education) and COVID-19 related beliefs (trust in government, perceived certainty of COVID-19 science, vaccination beliefs) affected our outcome variables (vaccination intention, perceived vaccine effectiveness, trust and confidence in the government official, worry, uncertainty, excitement and confidence). We conducted multiple linear regressions on the differences in each outcome variable between ratings before and after receiving conflicting information (see Table A1). As in our analyses in the main paper, those who received the certain announcement reported the greatest differences between before and after receiving conflicting information. Beliefs towards vaccination also had an effect across most variables, whereby those with more positive beliefs towards vaccination experienced greater differences between before and after receiving conflicting information. This suggests that people who have more positive vaccination beliefs are more likely to be disappointed after receiving conflicting information about vaccine effectiveness. Perhaps this is due to them having greater expectations of vaccine effectiveness and being more surprised once those expectations are not fulfilled.

Table A1: Effects of certainty, demographics and COVID-19 beliefs on differences in outcome before and after conflicting information

	Vaccine		Government		Emotions			
	Vaccination Intention	Effectiveness	Trust	Confidence	Worry	Uncertainty	Excitement	Confidence
Announcement certainty	*B*=-0.27 (0.08)**	*B*=-0.18 (0.08)*	*B*=-0.32 (0.09)***	*B*=-0.39 (0.10)***	*B*=0.23 (0.10)*	*B*=0.35 (0.11)**	*B*=-0.20 (0.09)*	*B*=-0.16 (0.09)
Age	*B*<0.01 (<0.01)	*B*<0.01 (<0.01)	*B*<0.01 (<0.01)	*B*=0.01 (<0.01)	*B*<0.01 (<0.01)	*B*<0.01 (<0.01)	*B*<0.01 (<0.01)	*B*=0.01 (<0.01)**
Gender	*B*=0.08 (0.09)	*B*=0.17 (0.08)*	*B*=0.22 (0.10)*	*B*=0.07 (0.10)	*B*=0.11 (0.10)	*B*=0.03 (0.12)	*B*=0.12 (0.10)	*B*=0.06 (0.10)
Education	*B*<0.01 (0.05)	*B*=-0.03 (0.04)	*B*=-0.05 (0.05)	*B*=0.01 (0.05)	*B*=-0.02 (0.05)	*B*=-0.10 (0.06)	*B*=-0.02 (0.05)	*B*=0.04 (0.05)
Trust in government	*B*=0.08 (0.05)	*B*=0.04 (0.04)	*B*=0.09 (0.05)	*B*=0.14 (0.05)**	*B*<0.01 (0.05)	*B*=-0.03 (0.06)	*B*=0.04 (0.05)	*B*=0.04 (0.05)
Science certainty	*B*=-0.01 (0.01)	*B*<0.01 (0.01)	*B*=0.01 (0.01)	*B*=-0.01 (0.01)	*B*=-0.01 (0.01)	*B*=-0.01 (0.02)	*B*<0.01 (0.01)	*B*=-0.01 (0.01)

Vaccine beliefs	*B*=0.01 (0.01)*	*B*=0.01 (0.01)*	*B*=0.02 (0.01)***	*B*=0.03 (0.01)***	*B*<0.01 (<0.01)	*B*<0.01 (<0.01)	*B*=0.02 (0.01)***	*B*=0.02 (0.01)***
Model	**F=3.01, R^2=6.2%****	**F=2.67, R^2=5.5%***	**F=7.46, R^2=14%*****	**F=8.66, R^2=15.9%*****	F=0.99, R^2=2.1%	F=1.80, R^2=3.8%	**F=4.36, R^2=8.7%*****	**F=5.69, R^2=9.1%*****

Note: Each outcome variable represents the difference in rating before and after receiving conflicting information. Predictor variables are announcement certainty (1=certain, 2=uncertain), age, gender (1=male, 2=female, 3=non-binary), education (1=GCSE or equivalent, 2=A-level or equivalent, 3=undergraduate degree, 4=postgraduate degree), trust in the UK government (scores range from 1-5), beliefs about the certainty of COVID-19 related science (scores range from 3-21), positive beliefs towards vaccination (scores range from 12-60). * refers to p<.05, ** p<.01, *** p<.001.
