# Supplementary material for: Negative consequences of failing to communicate uncertainties during a pandemic: an online randomised controlled trial on COVID-19 vaccines
Source: BMJ Open. 2022 Sep 7;12(9):e051352. doi: 10.1136/bmjopen-2021-051352 (PMC9453426; doi:10.1136/bmjopen-2021-051352)
Supplement: Author's manuscript [file bmjopen-2021-051352.draft_revisions.pdf]

BMJ Open is committed to open peer review. As part of this commitment we make the peer review history of every article we publish publicly available.

When an article is published we post the peer reviewers' comments and the authors' responses online. We also post the versions of the paper that were used during peer review. These are the versions that the peer review comments apply to.

The versions of the paper that follow are the versions that were submitted during the peer review process. They are not the versions of record or the final published versions. They should not be cited or distributed as the published version of this manuscript.

BMJ Open is an open access journal and the full, final, typeset and author-corrected version of record of the manuscript is available on our site with no access controls, subscription charges or pay-per-view fees (http://bmjopen.bmj.com).

If you have any questions on BMJ Open's open peer review process please email info.bmjopen@bmj.com

BMJ Open

The negative consequences of failing to communicate uncertainties during a pandemic: An online experiment on COVID-19 vaccines

Journal:	*BMJ Open*
Manuscript ID	bmjopen-2021-051352
Article Type:	Original research
Date Submitted by the Author:	17-Mar-2021
Complete List of Authors:	Batteux, Eleonore; University College London<br>Bilovich, Avri; University College London<br>Johnson, Samuel; University of Warwick<br>Tuckett, David; University College London
Keywords:	COVID-19, PUBLIC HEALTH, MEDICAL EDUCATION & TRAINING

SCHOLARONE™
Manuscripts

I, the Submitting Author has the right to grant and does grant on behalf of all authors of the Work (as defined in the below author licence), an exclusive licence and/or a non-exclusive licence for contributions from authors who are: i) UK Crown employees; ii) where BMJ has agreed a CC-BY licence shall apply, and/or iii) in accordance with the terms applicable for US Federal Government officers or employees acting as part of their official duties; on a worldwide, perpetual, irrevocable, royalty-free basis to BMJ Publishing Group Ltd ("BMJ") its licensees and where the relevant Journal is co-owned by BMJ to the co-owners of the Journal, to publish the Work in this journal and any other BMJ products and to exploit all rights, as set out in our licence.

The Submitting Author accepts and understands that any supply made under these terms is made by BMJ to the Submitting Author unless you are acting as an employee on behalf of your employer or a postgraduate student of an affiliated institution which is paying any applicable article publishing charge ("APC") for Open Access articles. Where the Submitting Author wishes to make the Work available on an Open Access basis (and intends to pay the relevant APC), the terms of reuse of such Open Access shall be governed by a Creative Commons licence – details of these licences and which Creative Commons licence will apply to this Work are set out in our licence referred to above.

Other than as permitted in any relevant BMJ Author's Self Archiving Policies, I confirm this Work has not been accepted for publication elsewhere, is not being considered for publication elsewhere and does not duplicate material already published. I confirm all authors consent to publication of this Work and authorise the granting of this licence.

For peer review only

The negative consequences of failing to communicate uncertainties during a pandemic: An online experiment on COVID-19 vaccines

Eleonore Batteux[1], Avri Bilovich[1], Samuel G. B. Johnson[2], David Tuckett[1],

[1]University College London, [2]University of Warwick

Corresponding author: Eleonore Batteux, Division of Psychology and Language Sciences, University College London, 1-19 Torrington Place, London, WC1E 7HB, UK, e.batteux@ucl.ac.uk

Word count: 5,009 words

For peer review only

Abstract

Objective. To examine the impact of communicating uncertainties relating to COVID-19 vaccine effectiveness on vaccination intention and trust after people are exposed to conflicting information.

Design. Experimental design where participants were randomly allocated to one of two groups.

Setting. Online.

Participants. 328 adults from a UK research panel.

Intervention. Participants received either certain or uncertain communications from a government representative about COVID-19 vaccine effectiveness, before receiving conflicting information about effectiveness.

Main outcome measures. Vaccination intention and trust in government.

Results. Compared to those who received the uncertain announcement, participants who received the certain announcement reported a greater loss of vaccination intention (d=0.34, 95% CI [0.12, 0.56], p=.002) and trust (d=0.34, 95% CI [0.12, 0.56], p=.002) after receiving conflicting information.

Conclusions. Communicating with certainty about COVID-19 vaccines reduces vaccination intention and trust if conflicting information arises, whereas communicating uncertainties can protect people from the negative impact of exposure to conflicting information.

Trial registration number. Open Science Framework: https://osf.io/c73px/

Keywords: uncertainty; health communication; trust; vaccine uptake; COVID-19

For peer review only - http://bmjopen.bmj.com/site/about/guidelines.xhtml

Strengths and limitations of this study

- This study provides experimental evidence of the benefits of communicating with uncertainty rather than certainty during a pandemic
- Participants were randomly allocated to receive either certain or uncertain hypothetical communications about COVID-19 vaccines
- Vaccination uptake was measured using a single-item measure of intention

No decision in healthcare comes without a degree of uncertainty. When recommending a treatment, a medical professional knows its effectiveness and possible side effects, along with their associated probabilities, what we call *risks*. But she may also be aware there is uncertainty surrounding that probability estimate, sometimes called *ambiguity* or *radical uncertainty*. This kind of uncertainty is particularly salient in a pandemic, where we often do not know enough about the effectiveness of treatments and policies to be confident of their outcomes. In the case of COVID-19, vaccine research is still underway to confirm their effectiveness and risks. There is even greater uncertainty relating to the impact of the vaccination programme on the pandemic more broadly. To what extent will vaccines reduce transmission? When will restrictions be lifted? Could new variants render vaccines ineffective? These questions are full of unknown parameters.

Despite the prevalence of uncertainty, there is a lack of consensus on how best to communicate it [1]. A first step towards it has been to investigate how patients respond to communications of uncertainty, both in terms of whether they understand it and how it affects their decision-making. This work has largely painted a negative picture of uncertainty, which has led to interrogations on how best to communicate it (if at all) [2]. We take a different approach in this paper, where we investigate the negative consequences of *failing* to communicate uncertainties. Are there times where, however difficult it may be to communicate uncertainties, doing so is better than hiding them? Does failing to communicate uncertainties backfire if people find out they exist and are exposed to conflicting information? We explore these questions in the context of COVID-19 vaccines by investigating how people respond to conflicting vaccine communications.

Communicating uncertainty in health

In this paper, we make a distinction between risk or probabilistic uncertainty (e.g. 20% chance of benefit from treatment) and uncertainty, or what can also be referred to as ambiguity.

For peer review only - http://bmjopen.bmj.com/site/about/guidelines.xhtml

Uncertainty can take various forms: imprecision (e.g. 10-30% chance of benefit from treatment), conflict (e.g. experts disagreeing), lack of information (e.g. insufficient evidence) [2]. All three of these are present during a novel pandemic such as COVID-19, so we consider them together in this paper.

Uncertainty is communicated to varying degrees across healthcare. Physicians mention some form of uncertainty in most of their patient encounters, although this tends to be in vague terms (e.g. 'There is a chance it will/won't work') [3]. They express verbal uncertainty (e.g. "I don't know or "It's not clear") in most clinic visits [4]. However, physicians are less likely to communicate uncertainty if they believe patients will have negative reactions to it, as they tend to believe [5]. Interventions designed to communicate information to patients often include quantitative risk estimates, but mentioning uncertainty tends to be the exception [1,6]. This usually takes the form of verbal uncertainty (e.g. "about" or "up to") and only a minority include numerical uncertainty (e.g. confidence intervals or ranges). This highlights the lack of consensus for how and when to communicate uncertainty in health.

We need to know how communicating uncertainty affects people's judgments and decisions in a public health context, which can be informed by research on patients. Firstly, there are concerns it reduces understanding [7]. Patients have difficulty acknowledging there are uncertainties associated to quantitative risk estimates [8]. This could be because people generally think science can provide certainty [9] and therefore interpret expressions of uncertainty as incompetence rather than an inevitable feature of science. Explaining why there is uncertainty might help to mitigate misunderstandings, which has been recommended when communicating uncertainty in general [10].

Secondly, uncertainty can have negative effects on patients, although this often depends on how it is communicated. Verbal expressions of uncertainty by doctors can lower patient confidence [11] and satisfaction [3,12]. Accompanying these by behaviors like positive talk and giving information can actually increase patient satisfaction [4], as can involving patients in decisions [3]. Behavioral expressions of uncertainty such as referring to a book or computer do not lower patient confidence [11] and can reduce the negative impact of verbal expressions of uncertainty [12]. However, behaviors such as less fluent speech and less eye contact can reduce trust [13]. Finally, numerical expressions of uncertainty (e.g. ranges) can reduce trust and credibility [7,14] and increase perceptions of risk and worry, although less so when communicated visually compared to textually [7,8,15]. However, ranges do not necessarily have detrimental effects in other domains, where the evidence is more mixed and often context-

For peer review only - http://bmjopen.bmj.com/site/about/guidelines.xhtml

dependent [16–18]. In addition, providing numerical information about risks and benefits makes patients less likely to overturn their decision in the face of conflicting information [19].

We focus on the effects of communicating uncertainty in public health, which present differences worth considering. Discussing uncertainty around numerical risk estimates may decrease perceived competence but also increase perceived honesty [9,20]. People report preferring to see precision in communications, but would rather uncertainties be disclosed if they exist [9]. This suggests that if people are aware that uncertainties exist they may be suspicious of communications which do not mention them. A previous study of particular interest here investigated how people respond to a government official announcing a vaccine during a hypothetical novel pandemic. Those who received uncertain communications reported lower vaccination intention due to lower perceived risk of the virus and vaccine effectiveness, accompanied by lower trust in the official [21]. This is not surprising as the communications they used were verbal and highly uncertain (e.g. "we are not sure exactly how effective it will be"). This is different to the COVID-19 context where we have more precise information, despite prevailing uncertainties and changing recommendations. People may also expect uncertainties and welcome their disclosure.

Parallels can be drawn between public health communication and science communication more broadly. In science communication, a lack of consensus is damaging whereas scientific uncertainty, such as ranges or a lack of evidence, is not and can have positive effects [22]. Those who perceive science as uncertain are more favorable to uncertainty, echoing findings that if people expect uncertainty they want it communicated [9]. Interestingly, those who have high trust in science more strongly support a policy as consensus between experts increases, whereas high consensus actually lowers support in those who have low trust in science, possibly because it looks like collusion [23]. This poses a challenge to public health communication during a crisis, where addresses to the nation can be less personalized than during physician consultations.

What if uncertainties are not communicated?

When uncertainties do exist, can ignoring them backfire and eventually lead to worse outcomes? The literature indicates there are advantages to not communicating uncertainties, but it does not address the consequences once people are confronted with information which seems to conflict with what they were communicated. There are many instances where this applies. If a physician tells a patient their risk of developing an illness is 10% and the patient later develops it, do they lose trust in the physician? If the physician had discussed uncertainties

For peer review only - http://bmjopen.bmj.com/site/about/guidelines.xhtml

surrounding that estimate, could that have mitigated a loss of trust? The same applies to a public health context, where a vaccine might be 90% effective against a virus but does not mean the vaccinated are certain they will not get infected. Crucially, in contexts where evidence is lacking, new evidence can arise which invalidates previous communications. Any detrimental effects could perhaps be attenuated by being clear about the quality of evidence from the outset. Although disclosing uncertainties might have negative effects initially, over time it could protect against the consequences of people experiencing undesirable outcomes or conflicting information, which we know is particularly damaging in science communication [22].

This question has been explored in other contexts, where findings tend to suggest that communicating uncertainty can be beneficial in the long term. In an intelligence context, when people are told a terrorist attack occurred and shown the forecasts, they find forecasters who communicated with ranges as more credible and less worthy of blame than those who communicated point estimates [24]. In a geological context, there is no evidence of a difference between point and range forecasts in terms of perceived correctness and loss of credibility after unlikely events occur [18]. In a financial investment context, when forecasts of future returns turn out to be incorrect, forecasters who communicated with confidence and precision are perceived as less trustworthy than those who acknowledged uncertainty [25]. Interestingly, this did not lead investors to lose confidence in and pull out of their investment, showing that they blame the forecaster for incorrect forecasts but not the object of the forecast. It is worth investigating whether this applies to a medical context, i.e. whether failing to communicate uncertainties has worse consequences for confidence in the communicator than in the object of the communication (e.g. a treatment or vaccine).

The present research

We examine how uncertain communications affect trust and vaccination intention over time. Specifically, we test whether communicating uncertainty about COVID-19 vaccines limits any loss of trust and vaccination intention after people receive conflicting information about their effectiveness. We focus on COVID-19 for two reasons. The first being that we urgently need to understand how to effectively communicate about COVID-19 vaccines to maximize uptake and ensure the successful rollout of the vaccination programme. Vaccine hesitancy is a particular concern, linked to a lack of trust [26]. Secondly, although we use hypothetical communications in our study, COVID-19 provides a real pandemic context that participants can relate to and have knowledge of. This differs from a previous study on

For peer review only - http://bmjopen.bmj.com/site/about/guidelines.xhtml

communicating vaccine uncertainty, which referred to a hypothetical virus participants had very little knowledge of [21]. Our hypotheses were preregistered on the Open Science Framework (OSF; https://osf.io/c73px/) and are as follows.

Hypothesis 1: We expect people are less favorable to getting vaccinated after receiving uncertain compared to certain vaccine communications. This is in keeping with the literature on how people respond to uncertain communications in health and public health. Our first main outcome variable is vaccination intention, which we expect to be lower following uncertain communications as found in a previous study [21]. We investigate whether this is accompanied by lower perceptions of vaccine effectiveness, as has been found previously [21], stronger avoidance emotions (e.g. worry) and weaker approach emotions (e.g. excitement). Indeed, we expect emotions to be crucial to people's decision-making in contexts of uncertainty [27]. Our second main outcome variable is how uncertainty affects trust in communicators, which is crucial to both vaccine uptake and compliance to guidelines during a pandemic [26,28]. Previous studies suggest trust should be lower [14,21].

Hypothesis 2: Once people receive information which conflicts with earlier communications, we expect those who initially received certain communications experience more negative effects compared to those who received uncertain communications. We posit that communicating uncertainty makes people more likely to expect information to change over time and therefore less surprised and disappointed when confronted to new and conflicting information, as has been found in the financial domain [25]. On the other hand, communicating with unwarranted certainty may be perceived as intentionally misleading. This would not be surprising in the context of COVID-19 in the UK where government overpromising has eroded trust [29]. We expect to see greater reductions in vaccination intention in those receiving certain communications, accompanied by greater reductions in trust, perceived vaccine effectiveness and approach emotions and a greater increase in avoidance emotions.

To test these hypotheses, we conducted a study in November 2020, before any COVID-19 vaccine announcement and effectiveness rates were widely communicated. We presented participants from the general UK population with a hypothetical vaccine announcement containing information about the vaccine's effectiveness. Some received information which conveyed certainty about the rate of effectiveness whereas others received information which conveyed uncertainty. Participants were then told that they find some new research on the vaccine's effectiveness, which is significantly lower than communicated in the announcement for both the certain and uncertain announcement. We compare participants' vaccination

For peer review only - http://bmjopen.bmj.com/site/about/guidelines.xhtml

intention, trust, perceived vaccine effectiveness and affective reactions after receiving the announcement to after receiving conflicting information.

Method

Design. Communication certainty (1-certain, 2-uncertain) was manipulated between-subjects. Participants were randomly allocated to the certain or uncertain communication condition via the Qualtrics survey platform randomization function.

Participants. 328 participants residing in the UK were recruited using Prolific, an online participant recruitment platform (https://www.prolific.co/). A sample of 328 was required to find a small effect (d=0.20) for Hypotheses 2a-e with a mixed model ANOVA with high power (>.95) and alpha level (<.05). This sample size also allows enough power to test Hypothesis 1 in accordance with existing findings. Participants were compensated for their time at a rate of £7.50 per hour. They were asked demographic questions (age, gender, level of education) and questions about COVID-19. Firstly, how much trust they currently have in the government's handling of the COVID-19 crisis on a 5-point scale (1-not at all, 5-a great deal). Secondly, how reliable, precise and consistent they perceive the science relating to COVID-19 on a 7-point scale (1-reliable/precise/consistent, 7-unreliable/imprecise/inconsistent). These were added to provide an overall score on their perception of the certainty of COVID-19 science. Finally, participants completed the Vaccination Attitudes Examination scale which provides an overall score of favorability to vaccination [30] on a 5-point scale (1-strongly disagree, 5-strongly agree). Participant characteristics can be found in Table 1.

Patient and public involvement. The public was involved in the development of the communications used in the study. We conducted an online pilot study with 50 UK participants to check that the communications about vaccine effectiveness were understandable and believable, with the opportunity for participants to provide feedback.

For peer review only

Table 1: Participant characteristics

Demographics	
Age	M=35.09 (SD=11.36)
Gender	28% Male
	71% Female
	1% Non-binary
Education	11% GCSE or equivalent

For peer review only - http://bmjopen.bmj.com/site/about/guidelines.xhtml

	23.5% A-level or equivalent
	45% Undergraduate degree
	20% Postgraduate degree
Beliefs	
Trust in gov	*M*=2.13 (*SD*=0.99)
Science certainty	*M*=11.47 (*SD*=4.10)
Vaccinations	*M*=39.97 (*SD*=10.02)

Note: Trust in government can range from 1-5, science certainty from 3-21, and vaccination attitudes from 12-60 (with higher figures indicating more favorable attitudes to vaccination).

For peer review only

Scenario. Participants were reminded they are in the middle of the COVID-19 pandemic and told to imagine they hear a public health government representative make a vaccine announcement on the news. This announcement states that a vaccine has passed the necessary checks and will soon be available. For those in the certain condition the representative says: "I can confirm that the vaccine is 60% effective. This means that, although the vaccine might not work for everyone, there is a very good chance that it will work for you. This vaccine will significantly drive down the infection rate and we will be able to remove the restrictive measures we put in place to combat the virus." In the uncertain condition the representative says: "The vaccine is between 50 and 70% effective. The reason I can't give a more precise estimate is because the data we have doesn't allow that. There might be some things we don't know yet about the vaccine, but this is the best available option. Although it might not work for everyone, there is a chance it will work for you. This vaccine will hopefully drive down the infection rate and we may be able to remove the restrictive measures we put in place to combat the virus." Then, all participants are told: "a week later, the vaccine is available and you can book an appointment with your local GP practice. Before deciding whether to get it, you want to read the research on the vaccine's effectiveness. You find the latest international piece of research which is deemed to have the most reliable data. This tells you that the vaccine is actually nearer to 40% effective."

Outcome measures. Measures were taken after participants received the initial announcement and after they read the additional research about the vaccine's effectiveness. Participants were asked how much confidence and trust they have in the government representative, how effective they think the vaccine is, how they feel about getting the vaccine (excited, confident, worried, uncertain) on 5-point scales (1-not at all, 5-a great deal) and how likely they are to get the vaccine on a 5-point scale (1-definitely not, 5-definitely yes).

For peer review only - http://bmjopen.bmj.com/site/about/guidelines.xhtml

Results

Our findings are broadly consistent across measures of vaccination intention, vaccine effectiveness, trust and confidence in government and emotion. They support the hypothesis that conflicting information leads to more negative effects among those who were exposed to certain compared to uncertain communications (Hypothesis 2). However, they do not support the hypothesis that people are initially more favorable to certain compared to uncertain communications (Hypothesis 1). We detail further analyses with demographics and Covid-19 related beliefs in the Supplementary File, which broadly do not affect our findings. All participants are included in our analyses.

Vaccination. The certain announcement led to a greater decline in vaccination intention following exposure to conflicting information (see Figure 1). Indeed, there was no difference in vaccination intention between people who received the certain and uncertain announcement after the announcement (t_{326}=-0.12, p=.903, d=0.01 95% CI [-0.20, 0.23]), but there was a marginal difference after reading the conflicting information (t_{326}=-1.804, p=.072, d=0.20 95% CI [0.02, 0.42]) ($F_{1,326}$=9.50, p=.002, η_p^2=0.03). In other words, those who received the certain announcement experienced a greater reduction in vaccination intention than those who received the uncertain announcement (t_{326}=3.08, p=.002, d=0.34 95% CI [0.12, 0.56]). Participants had stronger vaccination intentions after the announcement than after reading conflicting information ($F_{1,326}$=134.47, p<.001, η_p^2=0.29) and there was no overall difference between those receiving the certain and uncertain announcement ($F_{1,326}$=1.02, p=.314, η_p^2<0.01).

The pattern was the same for effectiveness, where the certain announcement led to a greater decline in perceived effectiveness (see Figure 1). Participants receiving the certain and uncertain announcement perceived the effectiveness equally after the announcement (t_{326}=0.06, p=.951, d=0.01 95% CI [-0.23, 0.21]), whereas those who received the certain announcement perceived it as less effective after reading conflicting information (t_{326}=-1.99, p=.048, d=0.22 95% CI [-0.00, 0.44]) ($F_{1,326}$=5.45, p=.020, η_p^2=0.02). Participants thought the vaccine was more effective after the announcement than after reading conflicting information ($F_{1,326}$=232.63, p<.001, η_p^2=0.42) and there was no overall significant difference between those receiving the certain and uncertain announcement ($F_{1,326}$=1, p=.318, η_p^2<0.01).

Government. The certain announcement led to a greater decline in trust and confidence in the government representative after exposure to conflicting information (see Figure 2). Both groups were equally trusting of the government representative after the announcement (t_{326}=-

For peer review only - http://bmjopen.bmj.com/site/about/guidelines.xhtml

0.54, p=.957, d=0.01 95% CI [-0.21, 0.22]), whereas those who received the certain announcement were less trusting after reading conflicting information (t_{326}=-3.04, p=.003, d=0.34 95% CI [0.12, 0.55]) ($F_{1,326}$=9.54, p=.002, η_p^2=0.03). In other words, those who received the certain announcement experienced a greater reduction in trust (t_{326}=3.09, p=.002, d=0.34 95% CI [0.12, 0.56]). Participants had more trust in the government representative after their announcement than after reading conflicting information ($F_{1,326}$=187.12, p<.001, η_p^2=0.37) and there was no overall significant difference between those receiving the certain and uncertain announcement ($F_{1,326}$=2.70, p=.101, η_p^2=0.01).

This was also the case for confidence (see Figure 2). Both groups were equally confident in the government representative after the announcement (t_{326}=0.79, p=.914, d=0.01 95% CI [-0.23, 0.21]), whereas those who received the certain announcement were less confident after reading conflicting information (t_{326}=-3.45, p=.001, d=0.38 95% CI [0.16, 0.60]) ($F_{1,326}$=12.08, p=.001, η_p^2=0.04). Indeed, those who received the certain announcement experienced a greater reduction in confidence (t_{326}=3.48, p=.001, d=0.38 95% CI [0.17, 0.60]). Participants were more confident in the government representative after their announcement than after reading conflicting information ($F_{1,326}$=170.61, p<.001, η_p^2=0.34) and there was no overall significant difference between those receiving the certain and uncertain announcement ($F_{1,326}$=3.13, p=.078, η_p^2=0.01).

Predictors of vaccination intention. In a previous study on communicating uncertainty about vaccines during a pandemic, perceived vaccine effectiveness mediated the relationship between communicated uncertainty and vaccination intention but trust in the government representative did not [21]. We explored whether this was also the case here using the PROCESS macro for SPSS [31] (see Figure 3). We find that both trust in the government representative (b=0.09, 95% CI[0.02,0.18]) and perceived effectiveness (b=0.14, 95% CI[0.003,0.29]) mediate the relationship between announcement certainty and vaccination intention. Participants who received the uncertain announcement were more likely to want to get vaccinated, both because they had higher trust in the government representative and because they perceived the vaccine as more effective after receiving conflicting information. Both of these mechanisms contribute to the effect of uncertainty communication on vaccination intention. Trust may not explain the effect of uncertainty communication on vaccination intention when the announcement is made [21], but it does here after participants are exposed to conflicting information.

For peer review only - http://bmjopen.bmj.com/site/about/guidelines.xhtml

Emotions. Although the pattern of findings on emotions is similar, the differences between those receiving the certain and uncertain announcement were less clear, perhaps due to the hypothetical nature of the study. The certain announcement led to a greater increase in avoidance emotions after exposure to conflicting information (see Figure 4). Participants were less worried after the announcement than after reading conflicting information ($F_{1,326}$=60.50, p<.001, η_p^2=0.16), which was qualified by an interaction with the certainty of the announcement ($F_{1,326}$=4.86, p=.028, η_p^2=0.02). Those who received the certain announcement experienced a greater increase in worry than those who received the uncertain announcement (t_{326}=-2.20, p=.028, d=0.24 95% CI [0.03, 0.46]), although there was no statistical difference between each group after receiving the announcement (t_{326}=-0.97, p=.332, d=0.11 95% CI [-0.11, 0.32]) or reading the conflicting information (t_{326}=0.51, p=.614, d=0.06 95% CI [-0.16, 0.27]). There was no overall significant difference between those receiving the certain and uncertain announcement ($F_{1,326}$=0.05, p=.819, η_p^2<0.01).

Participants were less uncertain after the announcement than after reading conflicting information ($F_{1,326}$=19.35, p<.001, η_p^2=0.06), which was qualified by an interaction with the certainty of the announcement ($F_{1,326}$=9.27, p=.003, η_p^2=0.03). Those who received the certain announcement experienced a greater increase in uncertainty than those who received the uncertain announcement (t_{326}=-3.05, p=.003, d=0.34 95% CI [0.12, 0.55]), although there was no statistical difference between each group after receiving the announcement (t_{326}=-1.70, p=.091, d=0.19 95% CI [-0.03, 0.40]) or reading the conflicting information (t_{326}=0.74, p=.462, d=0.08 95% CI [-0.14, 0.30]). There was no overall significant difference between those receiving the certain and uncertain announcement ($F_{1,326}$=0.24, p=.628, η_p^2<0.01).

We do not find that the certain announcement leads to a greater decrease in approach emotions after conflicting information (see Figure 4). Participants were more excited about the vaccine after the announcement than after reading conflicting information ($F_{1,326}$=127.76, p<.001, η_p^2=0.28) but the interaction with the certainty of the announcement was marginally significant ($F_{1,326}$=1.20, p=.060, η_p^2=0.01). There was no overall significant difference between those receiving the certain and uncertain announcement ($F_{1,326}$=1.05, p=.306, η_p^2<0.01). Participants were more confident about the vaccine after the announcement than after reading conflicting information ($F_{1,326}$=126.09, p<.001, η_p^2=0.28) but the interaction with the certainty of the announcement was not significant ($F_{1,326}$=2.16, p=.142, η_p^2=0.01). There was no overall difference between those receiving the certain and uncertain announcement ($F_{1,326}$=1.41, p=.235, η_p^2<0.01).

For peer review only - http://bmjopen.bmj.com/site/about/guidelines.xhtml

Discussion

Communicating uncertainties had protective effects against new conflicting information. Participants showed a greater reduction in vaccination intention after receiving information which conflicted with communications delivered with certainty, as opposed to communications which acknowledged uncertainties. This was accompanied by a greater reduction in trust in the communicator and perceived vaccine effectiveness, which both affected vaccination intention. Participants also experienced a greater increase in avoidance emotions (worry and uncertainty) following information which conflicted with certain as opposed to uncertain communications. We did not find a decline in approach emotions, although they were quite low to begin with.

The picture is more complicated when it comes to differences between certain and uncertain communications at each time point. At the time of the vaccine announcement, we do not find clear evidence that those who received uncertain communications are less likely to get vaccinated. This contrasts previous findings, although communications in those studies expressed greater uncertainty than in ours [21]. While most of the previous literature indicates that communicating uncertainty has damaging effects [2], our findings are an example of the kinds of contexts in which those effects might be weaker, i.e. when uncertainty is particularly salient. Patients might not expect scientific uncertainty generally [9], but people have been exposed to it during COVID-19 and may therefore expect it and want it communicated [22].

Once people receive information which conflicts with the vaccine announcement, we see differences between those exposed to the certain and uncertain announcement. Crucially, the government representative who delivered the announcement appears more trustworthy to those who were exposed to uncertainty. Communicating with unwarranted certainty damages trust, which echoes the finding that the UK government's overpromising during the COVID-19 pandemic eroded trust [29]. Those who received the certain announcement now perceive the vaccine as less effective, although the difference with vaccination intention is less clear. Having said that, those who experience a strong decline in trust and perceived vaccine effectiveness following the certain announcement also experience a strong decline in vaccination intention, making it weaker compared to those who received the uncertain announcement. Although communicating with certainty about vaccines is more damaging for trust in communicators than for vaccination intention, as findings in the financial domain suggested [25], the effects on vaccination intention remain a problem.

Limitations

For peer review only - http://bmjopen.bmj.com/site/about/guidelines.xhtml

Our findings highlight the benefits of communicating uncertainties in health, but they are only a starting point and we recommend caution in implementing these findings. We largely focused on uncertainties relating to vaccine effectiveness. Figures of vaccine effectiveness are often communicated with precision (e.g. 70% effectiveness) even though they come with confidence intervals, which we communicated in this study. There are many other uncertainties relating to vaccines during a novel pandemic worth exploring. Risks of side effects, including those not detectable in rapid trials, are particularly important to the public when making vaccination decisions [32]. Many are motivated to get vaccinated to reduce the spread of the virus and lift restrictions, but whether the vaccination programme can do so is not necessarily known from the outset [33]. It is unclear which of these uncertainties will have a stronger impact on vaccination intention, although we expect all of these to have similar effects to what we report here. Moreover, we only exposed participants to one instance of conflicting information, whereas there are likely to be more throughout a pandemic. Vaccination intention and trust are likely to evolve over time and may be more impacted by repeated exposures.

Given the hypothetical nature of our study, caution is warranted when applying findings. We used a hypothetical delay between the vaccine announcement and receiving conflicting information. This makes generalization to real instances more difficult, given that time delays increase the likelihood that people forget the information they receive and therefore do not interpret new information as conflicting with it. Having said that, government communications and new information are likely to be highly mediatized and conflicts made salient during a crisis like COVID-19. In addition, we used a real pandemic situation where participants had prior knowledge and relevant experiences. They are likely to have been more engaged and invested than in completely hypothetical studies.

Finally, it would be valuable to know how well these findings generalize beyond a pandemic context in the UK. It is worth investigating whether our findings generalize to other situations, such as physician-patient interactions where communicating uncertainty seems initially problematic but may have long-term benefits that have not been uncovered yet. Generalizing beyond the UK context would be valuable to inform global communication practices. Given that trust in government is important for vaccine uptake beyond the UK [26], we expect findings would be similar in other countries.

Implications for research and policy

Our findings highlight the negative consequences of failing to communicate uncertainties. Although communicating with certainty can initially have benefits, if that

For peer review only - http://bmjopen.bmj.com/site/about/guidelines.xhtml

certainty is not warranted it can have damaging consequences in the long run. Communicators should consider the quality of the evidence and whether people are likely to be exposed to diverging opinions and conflicting information. Anticipating this by discussing uncertainties could avoid negative consequences further down the line. In highly uncertain contexts, people may not actually be averse to uncertainties being communicated, unlike what previous studies in more certain contexts suggest [2]. More work is needed to establish whether people respond differently to uncertain communications depending on the level of contextual uncertainty.

How should we communicate uncertainties? Previous studies suggest some formats are more effective [8]. We used several ways of communicating uncertainty here, which at present we cannot tease apart. We manipulated the uncertainty of vaccine effectiveness, which was a point estimate in the certain announcement and a range in the uncertain announcement. Ranges may communicate uncertainty but they also increase worry and reduce understanding [7], suggesting that they alone are not sufficient. We accompanied the range by an explanation for the uncertainty, which could have enabled people to understand the uncertainty better. We included verbal descriptions of uncertainty regarding the broader risks and benefits of vaccination which may have increased people's perception of uncertainty, perhaps making them respond less negatively to conflicting information later on. Future research should evaluate these methods in isolation to better understand their relative effectiveness.

Who is best placed to communicate these uncertainties? Our study does not address this, although we provide the following reflections which could inform future research. People might have different expectations of government compared to medical practitioners. People have particularly low levels of trust in politicians [34]. The effects we find on trust could be due to participants perceiving the government representative as misleading them into getting vaccinated. People might have other expectations of medical practitioners, including certainty in their communications, thereby reacting negatively to expressions of uncertainty (although may react even more negatively if uncertainty that was not communicated is later revealed). Uncertainty could perhaps be interpreted as incompetence from medical practitioners but honesty from politicians, who have had a tendency to overpromise during the COVID-19 pandemic [29]. There may be instances where governments are better placed to communicate uncertainty, particularly during a national crisis, which further research should clarify.

Conclusion

During a novel pandemic, where evidence is lacking and evolves over time, people often face changing and conflicting information. Under these circumstances, we show that

For peer review only - http://bmjopen.bmj.com/site/about/guidelines.xhtml

communicating uncertainties attenuates the negative consequences of being faced with conflicting information. Although it comes with challenges, communicating uncertainty can be beneficial for maintaining trust and patient commitment over time. It takes more account of the potential for health care communications to develop active expertise in its recipients, thereby developing shared and resilient understanding [35,36]. Our findings support calls for greater transparency about uncertainty in communications relating to COVID-19 [37,38].

Acknowledgements

We thank Gerd Gigerenzer for comments on the final manuscript.

Author Contributions

The study was conceptualized by EB and AB. EB collected and analyzed the data. EB, AB, SJ and DT contributed to and approved the final manuscript. The corresponding author attests that all listed authors meet authorship criteria and that no others meeting the criteria have been omitted.

Funding Source

This research has been supported by the Think Forward Initiative (a partnership between ING Bank, Deloitte, Dell Technologies, Amazon Web Services, IBM, and the Center for Economic Policy Research – CEPR). Grant number not applicable. The views and opinions expressed in this paper are solely those of the authors and do not necessarily reflect the official policy or position of the Think Forward Initiative or any of its partners.

Competing interests

All authors declare: support from the Think Forward Initiative for the funding of the submitted work; no financial relationships with any organisations that might have an interest in the submitted work in the previous three years; no other relationships or activities that could appear to have influenced the submitted work.

Data availability

Anonymized data is available on Open Science Framework (https://osf.io/c73px/).

For peer review only - http://bmjopen.bmj.com/site/about/guidelines.xhtml

For peer review only

Ethics approval

Ethical approval for this study was obtained from University College London's Research Ethics Committee (approval number: 15313/001). Participants gave informed consent before taking part.

Transparency declaration

The lead author EB affirms that the manuscript is an honest, accurate, and transparent account of the study being reported; that no important aspects of the study have been omitted; and that any discrepancies from the study as planned (and, if relevant, registered) have been explained.

Dissemination

Dissemination to study participants and patient organisations is not applicable given the study is of interest to policy-makers and practitioners more than patients themselves.

For peer review only

For peer review only - http://bmjopen.bmj.com/site/about/guidelines.xhtml

References

1 Harrison M, Han PKJ, Rabin B, *et al.* Communicating uncertainty in cancer prognosis: A review of web-based prognostic tools. *Patient Educ Couns* 2019;**102**:842–9. doi:10.1016/j.pec.2018.12.009

2 Han PKJ, Babrow A, Hillen MA, *et al.* Uncertainty in health care: Towards a more systematic program of research. *Patient Educ Couns* 2019;**102**:1756–66. doi:10.1016/j.pec.2019.06.012

3 Politi MC, Clark MA, Ombao H, *et al.* Communicating uncertainty can lead to less decision satisfaction: A necessary cost of involving patients in shared decision making? *Heal Expect* 2010;**14**:84–91. doi:10.1111/j.1369-7625.2010.00626.x

4 Gordon GH, Joos SK, Byrne J. Physician expressions of uncertainty during patient encounters. *Patient Educ Couns* 2000;**40**:59–65. doi:10.1016/S0738-3991(99)00069-5

5 Portnoy DB, Han PKJ, Ferrer RA, *et al.* Physicians' attitudes about communicating and managing scientific uncertainty differ by perceived ambiguity aversion of their patients. *Heal Expect* 2011;**16**:362–72. doi:10.1111/j.1369-7625.2011.00717.x

6 Bansback N, Bell M, Spooner L, *et al.* Communicating Uncertainty in Benefits and Harms: A Review of Patient Decision Support Interventions. *Patient* 2017;**10**:311–9. doi:10.1007/s40271-016-0210-z

7 Longman T, Turner RM, King M, *et al.* The effects of communicating uncertainty in quantitative health risk estimates. *Patient Educ Couns* 2012;**89**:252–9. doi:10.1016/j.pec.2012.07.010

8 Han PKJ, Klein WMP, Lehman T, *et al.* Communication of uncertainty regarding individualized cancer risk estimates: Effects and influential factors. *Med Decis Mak* 2011;**31**:354–66. doi:10.1177/0272989X10371830

9 Johnson BB, Slovic P. Lay views on uncertainty in environmental health risk assessment. *J Risk Res* 1998;**1**:261–79. doi:10.1080/136698798377042

10 Van Der Bles AM, Van Der Linden S, Freeman ALJ, *et al.* Communicating uncertainty about facts, numbers and science. *R Soc Open Sci* 2019;**6**. doi:10.1098/rsos.181870

11 Ogden J, Fuks K, Gardner M, *et al.* Doctors expressions of uncertainty and patient confidence. *Patient Educ Couns* 2002;**48**:171–6. doi:10.1016/S0738-3991(02)00020-4

12 Johnson CG, Levenkron JC, Suchman AL, *et al.* Does physician uncertainty affect patient satisfaction? *J Gen Intern Med* 1988;**3**:144–9. doi:10.1007/BF02596120

13 Blanch-Hartigan D, van Eeden M, Verdam MGE, *et al.* Effects of communication

For peer review only

For peer review only - http://bmjopen.bmj.com/site/about/guidelines.xhtml

about uncertainty and oncologist gender on the physician-patient relationship. *Patient Educ Couns* 2019;**102**:1613–20. doi:10.1016/j.pec.2019.05.002

14 Schapira MM, Nattinger AB, McHorney CA. Frequency or probability? A qualitative study of risk communication formats used in health care. *Med Decis Mak* 2001;**21**:459–67. doi:10.1177/0272989X0102100604

15 Han PKJ, Klein WMP, Lehman TC, *et al.* Layperson's responses to the communication of uncertainty regarding cancer risk estimates. *Med Decis Mak* 2009;**29**:391–403. doi:10.1177/0272989X08327396

16 Batteux E, Bilovich A, Johnson S, *et al.* Impressed by numbers: The extent to which novice investors favor precise numerical information in a context of uncertainty. *SSRN Electron J* Published Online First: 2020. doi:10.2139/ssrn.3595409

17 van der Bles AM, van der Linden S, Freeman ALJ, *et al.* The effects of communicating uncertainty on public trust in facts and numbers. *Proc Natl Acad Sci U S A* 2020;**117**:7672–83. doi:10.1073/pnas.1913678117

18 Jenkins SC, Harris AJL, Lark RM. When unlikely outcomes occur: the role of communication format in maintaining communicator credibility. *J Risk Res* 2019;**22**:537–54. doi:10.1080/13669877.2018.1440415

19 Wegwarth O, Wagner GG, Gigerenzer G. Can facts trump unconditional trust? Evidence-based information halves the influence of physicians' non-evidence-based cancer screening recommendations. *PLoS One* 2017;**12**:1–12. doi:10.1371/journal.pone.0183024

20 Johnson BB, Slovic P. Presenting Uncertainty in Health Risk Assessment: Initial Studies of Its Effects on Risk Perception and Trust. *Risk Anal* 1995;**15**:485–94. doi:10.1111/j.1539-6924.1995.tb00341.x

21 Han PKJ, Zikmund-Fisher BJ, Duarte CW, *et al.* Communication of Scientific Uncertainty about a Novel Pandemic Health Threat: Ambiguity Aversion and Its Mechanisms. *J Health Commun* 2018;**23**:435–44. doi:10.1080/10810730.2018.1461961

22 Gustafson A, Rice RE. A review of the effects of uncertainty in public science communication. *Public Underst Sci* 2020;**29**:614–33. doi:10.1177/0963662520942122

23 Aklin M, Urpelainen J. Perceptions of scientific dissent undermine public support for environmental policy. *Environ Sci Policy* Published Online First: 2014. doi:10.1016/j.envsci.2013.10.006

24 Dieckmann NF, Mauro R, Slovic P. The effects of presenting imprecise probabilities

For peer review only - http://bmjopen.bmj.com/site/about/guidelines.xhtml

in intelligence forecasts. *Risk Anal* 2010;**30**:987–1001. doi:10.1111/j.1539-6924.2010.01384.x

25 Batteux E, Bilovich A, Johnson SGB, *et al.* When precision backfires: The effects of unwarranted precision on consumer loyalty. *Work Pap* 2020.

26 Lazarus J V, Ratzan S, Palayew A, *et al.* Hesitant or not? A global survey of potential acceptance of a COVID-19 vaccine Jeffrey. *MedRxiv* 2020;**preprint**:1–31. doi:10.1038/s41591-020-1124-9

27 Tuckett D, Nikolic M. The role of conviction and narrative in decision-making under radical uncertainty. *Theory Psychol* 2017;**27**:501–23. doi:10.1177/0959354317713158

28 Wright L, Steptoe A, Fancourt D. What predicts adherence to COVID-19 government guidelines ? Longitudinal analyses of 51 , 000 UK adults . *MedRxiv* 2020.

29 C19 National Foresight Group. Communications and the Covid-19 Pandemic. 2020.

30 Martin LR, Petrie KJ. Understanding the Dimensions of Anti-Vaccination Attitudes: the Vaccination Attitudes Examination (VAX) Scale. *Ann Behav Med* Published Online First: 2017. doi:10.1007/s12160-017-9888-y

31 Hayes AF. PROCESS: A versatile computational tool for observed variable mediation, moderation, and conditional process modeling. White Pap. 2012. doi:978-1-60918-230-4

32 Paul E, Steptoe A, Fancourt D. Attitudes towards vaccines and intention to vaccinate against COVID-19: Implications for public health communications. *Lancet Reg Heal - Eur* 2020;**1**. doi:10.1016/j.lanepe.2020.100012

33 Peiris M, Leung GM. What can we expect from first-generation COVID-19 vaccines? *Lancet* 2020;**6736**:20–1. doi:10.1016/s0140-6736(20)31976-0

34 Edelman. Edelman Trust Barometer 2020. Published Online First: 2020. doi:https://www.edelman.com/trustbarometer

35 Tuckett D. Explanatory models and conviction narratives. In: Christmas S, Michie S, West R, eds. *Thinking about Behaviour Change: An Interdisciplinary Dialogue*. Silverback Publishing 2015.

36 Tuckett D, Boulton M, Olson C, *et al. Meetings between experts: An approach to sharing ideas in medical consultations.* London and New York: : Tavistock Publications 1985.

37 Blastland M, Freeman ALJ, Van Der Linden S, *et al.* Five rules for evidence communication. *Nature* 2020;**587**. doi:10.1038/d41586-020-03189-1

38 Sunstein C, Altieri E, Kim M, *et al.* Behavioural considerations for acceptance and

For peer review only - http://bmjopen.bmj.com/site/about/guidelines.xhtml

uptake of COVID-19 vaccines. 2020.

For peer review only

For peer review only - http://bmjopen.bmj.com/site/about/guidelines.xhtml

Figure legends

Figure 1: Vaccination intention and perceived vaccine effectiveness before receiving conflicting information (i.e. after the vaccine announcement) and after receiving conflicting information by announcement certainty.

Figure 2: Trust and confidence in the government representative who made the vaccine announcement before receiving conflicting information (i.e. after the vaccine announcement) and after receiving conflicting information by announcement certainty.

Figure 3: Relationship between announcement certainty and vaccination intention after receiving conflicting information mediated by trust in government representative and perceived vaccine effectiveness. * refers to $p<.05$, ** refers to $p<.01$, *** refers to $p<.001$.

Figure 4: Emotions before receiving conflicting information (i.e. after the vaccine announcement) and after receiving conflicting information by announcement certainty.

For peer review only

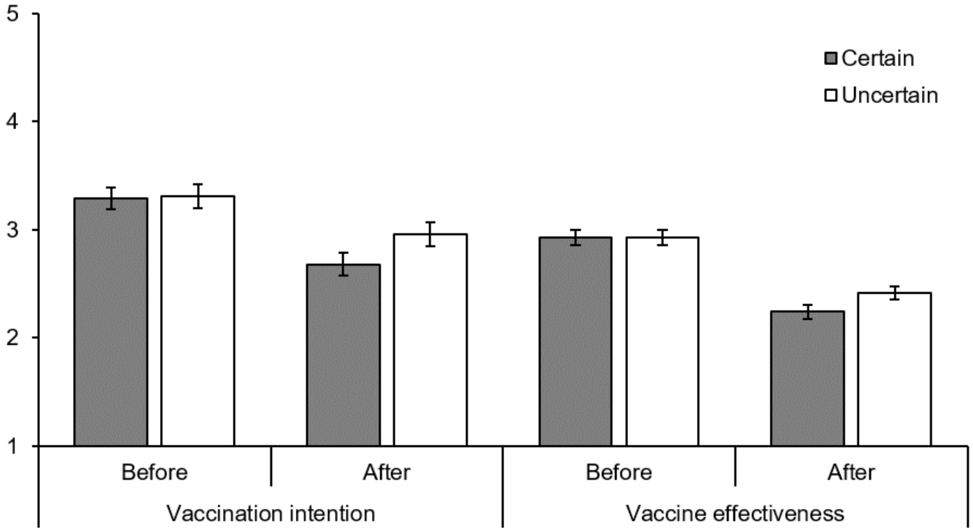

Figure 1: Vaccination intention and perceived vaccine effectiveness before receiving conflicting information (i.e. after the vaccine announcement) and after receiving conflicting information by announcement certainty.

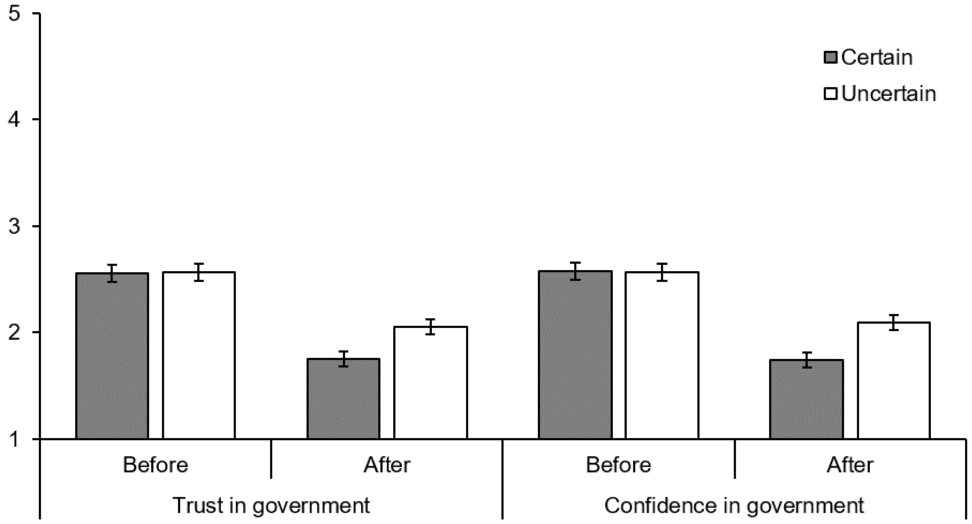

Figure 2: Trust and confidence in the government representative who made the vaccine announcement before receiving conflicting information (i.e. after the vaccine announcement) and after receiving conflicting information by announcement certainty.

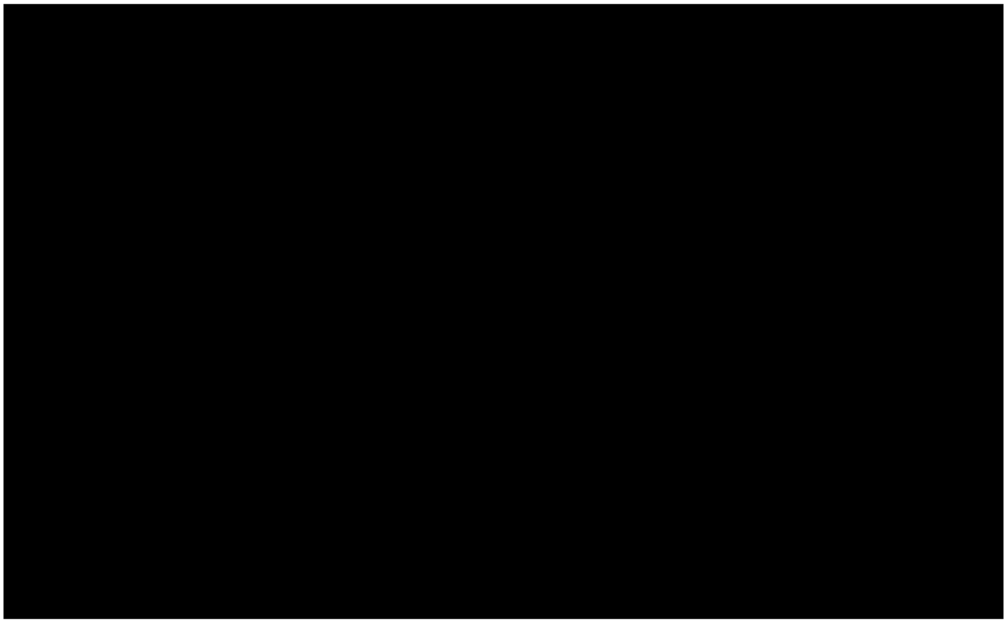

Figure 3: Relationship between announcement certainty and vaccination intention after receiving conflicting information mediated by trust in government representative and perceived vaccine effectiveness. * refers to p<.05, ** refers to p<.01, *** refers to p<.001.

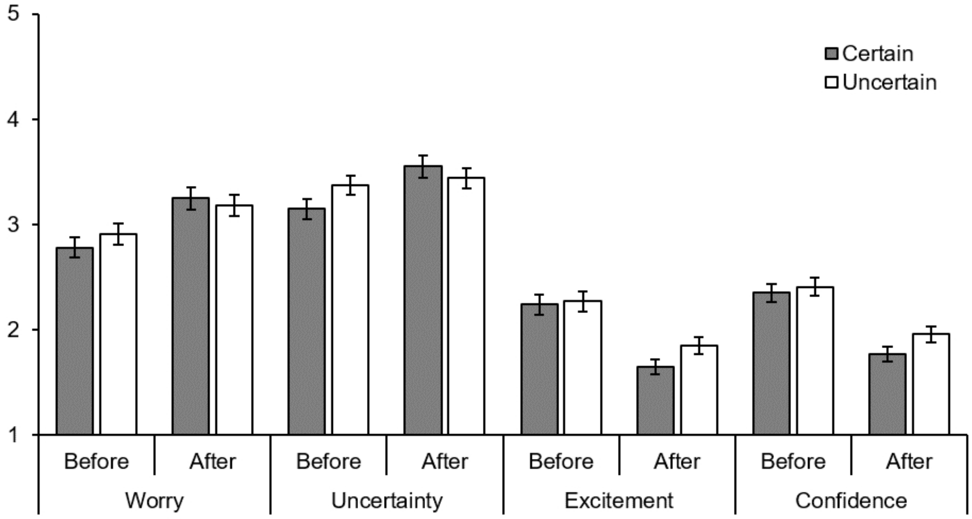

Figure 4: Emotions before receiving conflicting information (i.e. after the vaccine announcement) and after receiving conflicting information by announcement certainty.

Appendix: Further analyses on outcome variables

We investigated whether announcement certainty, demographics (age, gender, education) and COVID-19 related beliefs (trust in government, perceived certainty of COVID-19 science, vaccination beliefs) affected our outcome variables (vaccination intention, perceived vaccine effectiveness, trust and confidence in the government official, worry, uncertainty, excitement and confidence). We conducted multiple linear regressions on the differences in each outcome variable between ratings before and after receiving conflicting information (see Table A1). As in our analyses in the main paper, those who received the certain announcement reported the greatest differences between before and after receiving conflicting information. Beliefs towards vaccination also had an effect across most variables, whereby those with more positive beliefs towards vaccination experienced greater differences between before and after receiving conflicting information. This suggests that people who have more positive vaccination beliefs are more likely to be disappointed after receiving conflicting information about vaccine effectiveness. Perhaps this is due to them having greater expectations of vaccine effectiveness and being more surprised once those expectations are not fulfilled.

Table A1: Effects of certainty, demographics and COVID-19 beliefs on differences in outcome before and after conflicting information

	Vaccine		Government		Emotions			
	Vaccination Intention	Effectiveness	Trust	Confidence	Worry	Uncertainty	Excitement	Confidence
Announcement certainty	*B*=-0.27 (0.08)**	*B*=-0.18 (0.08)*	*B*=-0.32 (0.09)***	*B*=-0.39 (0.10)***	*B*=0.23 (0.10)*	*B*=0.35 (0.11)**	*B*=-0.20 (0.09)*	*B*=-0.16 (0.09)
Age	*B*<0.01 (<0.01)	*B*<0.01 (<0.01)	*B*<0.01 (<0.01)	*B*=0.01 (<0.01)	*B*<0.01 (<0.01)	*B*<0.01 (<0.01)	*B*<0.01 (<0.01)	*B*=0.01 (<0.01)**
Gender	*B*=0.08 (0.09)	*B*=0.17 (0.08)*	*B*=0.22 (0.10)*	*B*=0.07 (0.10)	*B*=0.11 (0.10)	*B*=0.03 (0.12)	*B*=0.12 (0.10)	*B*=0.06 (0.10)
Education	*B*<0.01 (0.05)	*B*=-0.03 (0.04)	*B*=-0.05 (0.05)	*B*=0.01 (0.05)	*B*=-0.02 (0.05)	*B*=-0.10 (0.06)	*B*=-0.02 (0.05)	*B*=0.04 (0.05)
Trust in government	*B*=0.08 (0.05)	*B*=0.04 (0.04)	*B*=0.09 (0.05)	*B*=0.14 (0.05)**	*B*<0.01 (0.05)	*B*=-0.03 (0.06)	*B*=0.04 (0.05)	*B*=0.04 (0.05)
Science certainty	*B*=-0.01 (0.01)	*B*<0.01 (0.01)	*B*=0.01 (0.01)	*B*=-0.01 (0.01)	*B*=-0.01 (0.01)	*B*=-0.01 (0.02)	*B*<0.01 (0.01)	*B*=-0.01 (0.01)

Vaccine beliefs	B=0.01 (0.01)*	B=0.01 (0.01)*	B=0.02 (0.01)***	B=0.03 (0.01)***	B<0.01 (<0.01)	B<0.01 (<0.01)	B=0.02 (0.01)***	B=0.02 (0.01)***
Model	**F=3.01, R^2=6.2%****	**F=2.67, R^2=5.5%***	**F=7.46, R^2=14%*****	**F=8.66, R^2=15.9%*****	F=0.99, R^2=2.1%	F=1.80, R^2=3.8%	**F=4.36, R^2=8.7%*****	**F=5.69, R^2=9.1%*****

Note: Each outcome variable represents the difference in rating before and after receiving conflicting information. Predictor variables are announcement certainty (1=certain, 2=uncertain), age, gender (1=male, 2=female, 3=non-binary), education (1=GCSE or equivalent, 2=A-level or equivalent, 3=undergraduate degree, 4=postgraduate degree), trust in the UK government (scores range from 1-5), beliefs about the certainty of COVID-19 related science (scores range from 3-21), positive beliefs towards vaccination (scores range from 12-60). * refers to p<.05, ** p<.01, *** p<.001.

For peer review only

CONSORT 2010 checklist of information to include when reporting a randomised trial*

Section/Topic	Item No	Checklist item	Reported on page No
**Title and abstract**			
	1a	Identification as a randomised trial in the title	1
	1b	Structured summary of trial design, methods, results, and conclusions (for specific guidance see CONSORT for abstracts)	2
**Introduction**			
Background and objectives	2a	Scientific background and explanation of rationale	1-4
	2b	Specific objectives or hypotheses	4-5
**Methods**			
Trial design	3a	Description of trial design (such as parallel, factorial) including allocation ratio	7
	3b	Important changes to methods after trial commencement (such as eligibility criteria), with reasons	NA
Participants	4a	Eligibility criteria for participants	7
	4b	Settings and locations where the data were collected	7
Interventions	5	The interventions for each group with sufficient details to allow replication, including how and when they were actually administered	9
Outcomes	6a	Completely defined pre-specified primary and secondary outcome measures, including how and when they were assessed	9
	6b	Any changes to trial outcomes after the trial commenced, with reasons	NA
Sample size	7a	How sample size was determined	7-8
	7b	When applicable, explanation of any interim analyses and stopping guidelines	NA
Randomisation:			
Sequence generation	8a	Method used to generate the random allocation sequence	7
	8b	Type of randomisation; details of any restriction (such as blocking and block size)	7
Allocation concealment mechanism	9	Mechanism used to implement the random allocation sequence (such as sequentially numbered containers), describing any steps taken to conceal the sequence until interventions were assigned	7
Implementation	10	Who generated the random allocation sequence, who enrolled participants, and who assigned participants to interventions	7
Blinding	11a	If done, who was blinded after assignment to interventions (for example, participants, care providers, those	7

For peer review only - http://bmjopen.bmj.com/site/about/guidelines.xhtml

		assessing outcomes) and how	
	11b	If relevant, description of the similarity of interventions	NA
Statistical methods	12a	Statistical methods used to compare groups for primary and secondary outcomes	9-14
	12b	Methods for additional analyses, such as subgroup analyses and adjusted analyses	9-14
**Results**			
Participant flow (a diagram is strongly recommended)	13a	For each group, the numbers of participants who were randomly assigned, received intended treatment, and were analysed for the primary outcome	7
	13b	For each group, losses and exclusions after randomisation, together with reasons	NA
Recruitment	14a	Dates defining the periods of recruitment and follow-up	7
	14b	Why the trial ended or was stopped	7
Baseline data	15	A table showing baseline demographic and clinical characteristics for each group	8
Numbers analysed	16	For each group, number of participants (denominator) included in each analysis and whether the analysis was by original assigned groups	9
Outcomes and estimation	17a	For each primary and secondary outcome, results for each group, and the estimated effect size and its precision (such as 95% confidence interval)	9-14
	17b	For binary outcomes, presentation of both absolute and relative effect sizes is recommended	NA
Ancillary analyses	18	Results of any other analyses performed, including subgroup analyses and adjusted analyses, distinguishing pre-specified from exploratory	12
Harms	19	All important harms or unintended effects in each group (for specific guidance see CONSORT for harms)	NA
**Discussion**			
Limitations	20	Trial limitations, addressing sources of potential bias, imprecision, and, if relevant, multiplicity of analyses	15-16
Generalisability	21	Generalisability (external validity, applicability) of the trial findings	15-16
Interpretation	22	Interpretation consistent with results, balancing benefits and harms, and considering other relevant evidence	15
**Other information**			
Registration	23	Registration number and name of trial registry	6
Protocol	24	Where the full trial protocol can be accessed, if available	6
Funding	25	Sources of funding and other support (such as supply of drugs), role of funders	18

*We strongly recommend reading this statement in conjunction with the CONSORT 2010 Explanation and Elaboration for important clarifications on all the items. If relevant, we also recommend reading CONSORT extensions for cluster randomised trials, non-inferiority and equivalence trials, non-pharmacological treatments, herbal interventions, and pragmatic trials. Additional extensions are forthcoming: for those and for up to date references relevant to this checklist, see www.consort-statement.org.

For peer review only - http://bmjopen.bmj.com/site/about/guidelines.xhtml

BMJ Open

The negative consequences of failing to communicate uncertainties during a pandemic: An online randomized controlled trial on COVID-19 vaccines

Journal:	*BMJ Open*
Manuscript ID	bmjopen-2021-051352.R1
Article Type:	Original research
Date Submitted by the Author:	31-Mar-2022
Complete List of Authors:	Batteux, Eleonore; University College London<br>Bilovich, Avri; University College London<br>Johnson, Samuel; University of Warwick<br>Tuckett, David; University College London
**Primary Subject Heading**:	Public health
Secondary Subject Heading:	Health policy
Keywords:	COVID-19, PUBLIC HEALTH, MEDICAL EDUCATION & TRAINING, Health policy < HEALTH SERVICES ADMINISTRATION & MANAGEMENT

SCHOLARONE™
Manuscripts

I, the Submitting Author has the right to grant and does grant on behalf of all authors of the Work (as defined in the below author licence), an exclusive licence and/or a non-exclusive licence for contributions from authors who are: i) UK Crown employees; ii) where BMJ has agreed a CC-BY licence shall apply, and/or iii) in accordance with the terms applicable for US Federal Government officers or employees acting as part of their official duties; on a worldwide, perpetual, irrevocable, royalty-free basis to BMJ Publishing Group Ltd ("BMJ") its licensees and where the relevant Journal is co-owned by BMJ to the co-owners of the Journal, to publish the Work in this journal and any other BMJ products and to exploit all rights, as set out in our licence.

The Submitting Author accepts and understands that any supply made under these terms is made by BMJ to the Submitting Author unless you are acting as an employee on behalf of your employer or a postgraduate student of an affiliated institution which is paying any applicable article publishing charge ("APC") for Open Access articles. Where the Submitting Author wishes to make the Work available on an Open Access basis (and intends to pay the relevant APC), the terms of reuse of such Open Access shall be governed by a Creative Commons licence – details of these licences and which Creative Commons licence will apply to this Work are set out in our licence referred to above.

Other than as permitted in any relevant BMJ Author's Self Archiving Policies, I confirm this Work has not been accepted for publication elsewhere, is not being considered for publication elsewhere and does not duplicate material already published. I confirm all authors consent to publication of this Work and authorise the granting of this licence.

For peer review only

The negative consequences of failing to communicate uncertainties during a pandemic: An online randomized controlled trial on COVID-19 vaccines

Eleonore Batteux[1], Avri Bilovich[1], Samuel G. B. Johnson[2], David Tuckett[1],
[1]University College London, [2]University of Warwick

Corresponding author: Eleonore Batteux, Division of Psychology and Language Sciences, University College London, 1-19 Torrington Place, London, WC1E 7HB, UK, e.batteux@ucl.ac.uk

Word count: 4,305 words

For peer review only

Abstract

Objective. To examine the impact of communicating uncertainties relating to COVID-19 vaccine effectiveness on vaccination intention and trust after people are exposed to conflicting information.

Design. Experimental design where participants were randomly allocated to one of two groups.

Setting. Online.

Participants. 328 adults from a UK research panel.

Intervention. Participants received either certain or uncertain communications from a government representative about COVID-19 vaccine effectiveness, before receiving conflicting information about effectiveness.

Main outcome measures. Vaccination intention and trust in government.

Results. Compared to those who received the uncertain announcement, participants who received the certain announcement reported a greater loss of vaccination intention (d=0.34, 95% CI [0.12, 0.56], p=.002) and trust (d=0.34, 95% CI [0.12, 0.56], p=.002) after receiving conflicting information.

Conclusions. Communicating with certainty about COVID-19 vaccines reduces vaccination intention and trust if conflicting information arises, whereas communicating uncertainties can protect people from the negative impact of exposure to conflicting information.

Trial registration number. Open Science Framework: https://osf.io/c73px/

Keywords: uncertainty; health communication; trust; vaccine uptake; COVID-19

For peer review only - http://bmjopen.bmj.com/site/about/guidelines.xhtml

Strengths and limitations of this study

- This study provides experimental evidence of the benefits of communicating with uncertainty rather than certainty during a pandemic.
- Participants were randomly allocated to receive either certain or uncertain hypothetical communications about COVID-19 vaccines.
- Vaccination uptake was measured using a single-item measure of intention.

No decision in healthcare comes without a degree of uncertainty. When recommending a treatment, a medical professional generally knows its effectiveness and possible side effects, along with their associated probabilities, i.e. *risks*. They may also be aware there is uncertainty surrounding that probability estimate, sometimes called *ambiguity* or *radical uncertainty*. This kind of uncertainty is particularly salient in a pandemic, where the precise outcomes of treatments and policies cannot be known. Earlier on in the COVID-19 vaccine roll out, research was still underway to confirm vaccines' effectiveness and risks. Accounts of damaging side effects, such as thrombosis following the AstraZeneca vaccine, severely damaged trust [1]. Today, there remain uncertainties about the effectiveness of vaccines against new variants.

Despite the prevalence of uncertainty, there is a lack of consensus on how best to communicate it [2]. A first step has been to investigate how patients respond to communications of uncertainty, which has largely uncovered negative impacts and led to interrogations on how best to communicate it (if at all) [3]. We take a different approach in this paper, where we investigate the negative consequences of *failing* to communicate uncertainties. Are there times where, however difficult it may be to communicate uncertainties, doing so is better than hiding them? Does failing to communicate uncertainties backfire if people find out they exist and are exposed to conflicting information? We explore these questions by investigating how people respond to conflicting COVID-19 vaccine communications.

Communicating uncertainty in health

In this paper, we distinguish risk or probabilistic uncertainty (e.g. 20% chance of benefit from treatment) from uncertainty, or what can also be referred to as ambiguity. Uncertainty can take various forms: imprecision (e.g. 10-30% chance of benefit from treatment), conflict (e.g. experts disagreeing), lack of information (e.g. insufficient evidence) [3]. All three are present during a pandemic like COVID-19, so we consider them together here.

For peer review only - http://bmjopen.bmj.com/site/about/guidelines.xhtml

Uncertainty is communicated to varying degrees across healthcare. Physicians mention some form of uncertainty in most of their patient encounters, although this tends to be in vague terms (e.g. 'There is a chance it will/won't work') [4] [5]. However, physicians are less likely to report that they would communicate uncertainty if they believe patients will have negative reactions to it, which tends to be the case [6]. Interventions designed to communicate information to patients often include quantitative risk estimates, but mentioning uncertainty tends to be the exception [2,7]. When mentioned, it is usually verbally (e.g. "about" or "up to"). This highlights the lack of consensus for how and when to communicate uncertainty in health.

This is not surprising given that uncertainty can have negative effects on patients, for both significant (e.g. cancer, [4]) and more minor (e.g. acne [8]) illnesses. Verbal expressions of uncertainty by doctors can lower patient confidence [8] and satisfaction [4,9]. Numerical expressions of uncertainty (e.g. ranges) can reduce trust and credibility [10,11] and increase perceptions of risk and worry, although less so when communicated visually compared to textually [11–13]. This could be because people generally think science can provide certainty [14] and therefore interpret expressions of uncertainty as signs of incompetence rather than an inevitable feature of science. Explaining why there is uncertainty might help to mitigate misunderstandings, which has been recommended when communicating uncertainty in general [15]. In addition, providing numerical information about risks and benefits makes patients less likely to overturn their decision in the face of conflicting information [16].

We focus on the effects of communicating uncertainty in public health, which present notable differences. Discussing uncertainty around numerical risk estimates may decrease perceived competence but also increase perceived honesty [14,17]. Although people report preferring to see precision in communications, they would rather uncertainties be disclosed if they exist [14]. This suggests that if people are aware that uncertainties exist, they may be suspicious of communications which do not mention them. Nonetheless, a previous study on vaccine communications during a hypothetical novel pandemic found that uncertain communications led to lower vaccination intention and lower trust in the communicator [18]. However, this may be because the communications were verbal and highly uncertain (e.g. "we are not sure exactly how effective it will be"). There is more precise information in the context of COVID-19, despite prevailing uncertainties.

What if uncertainties are not communicated?

When uncertainties do exist, can ignoring them backfire? The literature indicates there are advantages to not communicating uncertainties, but it does not address the consequences

For peer review only - http://bmjopen.bmj.com/site/about/guidelines.xhtml

once people are confronted with information which conflicts with what they were communicated. There are many instances where this applies. A vaccine might be 70% effective against infection, but that does not mean the vaccinated are certain they will not get infected. In contexts where evidence is lacking, new evidence can arise which invalidates previous communications. Although disclosing uncertainties might have negative effects initially, over time it could protect against the consequences of people experiencing undesirable outcomes or conflicting information, which is damaging in science communication [19].

In other contexts, communicating uncertainty can be beneficial in the long term. In an intelligence context, when people are told a terrorist attack occurred and shown the forecasts, they find forecasters who communicated uncertainty more credible and less worthy of blame [20]. In a geological context, there is no evidence of a difference between certain and uncertain forecasts in terms of perceived correctness and loss of credibility after unlikely events occur [21]. In a financial investment context, when forecasts of future returns turn out to be incorrect, forecasters who did not acknowledge uncertainty were perceived as less trustworthy [22]. Interestingly, this did not lead investors to pull out of their investment, showing that they blame the forecaster for incorrect forecasts but not the object of the forecast. It is worth investigating whether this applies to a medical context, i.e. whether failing to communicate uncertainties has worse consequences for confidence in the communicator than in the object of the communication (e.g. a treatment or vaccine).

The present research

We examine how uncertain communications affect trust and vaccination intention over time. Specifically, we test whether communicating uncertainty about COVID-19 vaccines limits any loss of trust and vaccination intention after people receive conflicting information about their effectiveness. We focus on COVID-19 given the need to maximize vaccine uptake, where low trust has been linked to vaccine hesitancy [23]. In addition, COVID-19 provides a real pandemic context that participants can relate to and have knowledge of. Our hypotheses were preregistered on the Open Science Framework [24] and are as follows.

Hypothesis 1: We expect people are less favorable to vaccination after receiving uncertain compared to certain communications. The first main outcome variable is vaccination intention, which we expect to be lower following uncertain communications, as found in a previous study [18]. We investigate whether this is accompanied by lower perceptions of vaccine effectiveness [18], stronger avoidance emotions (e.g. worry) and weaker approach

For peer review only - http://bmjopen.bmj.com/site/about/guidelines.xhtml

emotions (e.g. excitement). Indeed, emotions are crucial to decision-making in contexts of uncertainty [25]. The second main outcome variable is trust in communicators, which is crucial to both vaccine uptake and compliance to guidelines during a pandemic [23,26]. Previous studies suggest trust should be lower when uncertainty is communicated [10,18].

Hypothesis 2: Once people receive information which conflicts with earlier communications, we expect those who initially received certain communications to experience more negative effects compared to those who received uncertain communications. We posit that communicating uncertainty makes people more likely to expect information to change over time and therefore less affected when confronted to conflicting information. On the other hand, communicating with unwarranted certainty may be perceived as intentionally misleading. We expect to see greater reductions in vaccination intention in those receiving certain communications.

We conducted a study in November 2020, before COVID-19 effectiveness rates were widely communicated. We presented participants from the general UK population with a hypothetical vaccine announcement containing information about the vaccine's effectiveness, which either conveyed certainty or uncertainty. Participants were then given new information about vaccine effectiveness, which conflicts with the earlier announcement. We compare participants' vaccination intention, trust, perceived vaccine effectiveness and affective reactions after receiving the announcement to after receiving conflicting information.

Method

Design. Communication certainty (1-certain, 2-uncertain) was manipulated between-subjects. Participants were randomly allocated to the certain or uncertain communication condition via the Qualtrics survey platform randomization function and were blind to the condition they were allocated to.

Participants. 328 participants residing in the UK were recruited using Prolific, an online participant recruitment platform (https://www.prolific.co/) (see Figure 1). A sample of 328 was required to find a small effect (d=0.20) for Hypotheses 2a-e with a mixed model ANOVA with high power (>.95) and alpha level (<.05). This sample size also allows enough power to test Hypothesis 1 in accordance with existing findings. Participants were compensated for their time at a rate of £7.50 per hour. They were asked demographic questions (age, gender, level of education). They were then asked questions about COVID-19; firstly, how much trust they currently have in the government's handling of the COVID-19 crisis on a 5-point scale (1-not at all, 5-a great deal). Secondly, how reliable, precise and consistent they perceive the science

For peer review only - http://bmjopen.bmj.com/site/about/guidelines.xhtml

relating to COVID-19 on a 7-point scale (1-reliable/precise/consistent, 7-unreliable/imprecise/inconsistent). These were added to provide an overall score on their perception of the certainty of COVID-19 science. Finally, participants completed the Vaccination Attitudes Examination scale which provides an overall score of favorability to vaccination [27] on a 5-point scale (1-strongly disagree, 5-strongly agree). Participant characteristics can be found in Table 1.

Patient and public involvement. The public was involved in the development of the communications used in the study. We conducted an online pilot study with 50 UK participants to check that the communications about vaccine effectiveness were understandable and believable, with the opportunity for participants to provide feedback.

Table 1: Participant characteristics

Demographics	
Age	*M*=35.09 (*SD*=11.36)
Gender	28% Male
	71% Female
	1% Non-binary
Education	11% GCSE or equivalent
	23.5% A-level or equivalent
	45% Undergraduate degree
	20% Postgraduate degree
Beliefs	
Trust in gov	*M*=2.13 (*SD*=0.99)
Science certainty	*M*=11.47 (*SD*=4.10)
Vaccinations	*M*=39.97 (*SD*=10.02)

Note: Trust in government can range from 1-5, science certainty from 3-21, and vaccination attitudes from 12-60 (with higher figures indicating more favorable attitudes to vaccination).

Scenario. Participants were reminded they are in the middle of the COVID-19 pandemic and told to imagine they hear a public health government representative make a vaccine announcement on the news. This announcement states that a vaccine has passed the necessary checks and will soon be available. For those in the certain condition the representative says: "I can confirm that the vaccine is 60% effective. This means that, although the vaccine might not work for everyone, there is a very good chance that it will work for you. This vaccine will

For peer review only - http://bmjopen.bmj.com/site/about/guidelines.xhtml

significantly drive down the infection rate and we will be able to remove the restrictive measures we put in place to combat the virus." In the uncertain condition the representative says: "The vaccine is between 50 and 70% effective. The reason I can't give a more precise estimate is because the data we have doesn't allow that. There might be some things we don't know yet about the vaccine, but this is the best available option. Although it might not work for everyone, there is a chance it will work for you. This vaccine will hopefully drive down the infection rate and we may be able to remove the restrictive measures we put in place to combat the virus." Then, all participants are told: "a week later, the vaccine is available and you can book an appointment with your local GP practice. Before deciding whether to get it, you want to read the research on the vaccine's effectiveness. You find the latest international piece of research which is deemed to have the most reliable data. This tells you that the vaccine is actually nearer to 40% effective."

Outcome measures. Measures were taken after participants received the initial announcement and after they read the additional research about the vaccine's effectiveness. Participants were asked how much confidence and trust they have in the government representative, how effective they think the vaccine is, how they feel about getting the vaccine (excited, confident, worried, uncertain) on 5-point scales (1-not at all, 5-a great deal) and how likely they are to get the vaccine on a 5-point scale (1-definitely not, 5-definitely yes).

Analysis. As specified in the preregistered analysis plan, Hypotheses 1 and 2 were tested with mixed model ANOVAs. Announcement certainty (1-certain; 2-uncertain) was a between subjects factor and time point (1-after announcement; 2-after conflicting information) was a within subjects factor. This analysis was conducted for all dependent measures (vaccination intention, effectiveness, trust, confidence, emotions). Outcome assessors were not blind to the treatment group participants were allocated to.

Results

The findings are broadly consistent across measures of vaccination intention, vaccine effectiveness, trust and confidence in government and emotion. They support the hypothesis that conflicting information leads to more negative effects among those who were exposed to certain compared to uncertain communications (Hypothesis 2). However, they do not support the hypothesis that people are initially more favorable to certain compared to uncertain communications (Hypothesis 1). Further analyses with demographics and Covid-19 related beliefs are detailed in the Supplementary File, which broadly do not affect our findings.

For peer review only - http://bmjopen.bmj.com/site/about/guidelines.xhtml

Vaccination. The certain announcement led to a greater decline in vaccination intention following exposure to conflicting information (see Figure 2). There was no difference in vaccination intention between people who received the certain and uncertain announcement after the announcement (t_{326}=-0.12, p=.903, d=0.01 95% CI [-0.20, 0.23]), but there was a marginal difference after reading the conflicting information (t_{326}=-1.804, p=.072, d=0.20 95% CI [0.02, 0.42]) ($F_{1,326}$=9.50, p=.002, η_p^2=0.03). The significant interaction indicates that those who received the certain announcement experienced a greater reduction in vaccination intention than those who received the uncertain announcement. Participants had stronger vaccination intentions after the announcement than after reading conflicting information ($F_{1,326}$=134.47, p<.001, η_p^2=0.29) and there was no overall difference between those receiving the certain and uncertain announcement ($F_{1,326}$=1.02, p=.314, η_p^2<0.01).

The pattern was the same for effectiveness, where the certain announcement led to a greater decline in perceived effectiveness (see Figure 2). After the announcement, perceptions of effectiveness were comparable between those who received the certain and uncertain announcement (t_{326}=0.06, p=.951, d=0.01 95% CI [-0.23, 0.21]), whereas those who received the certain announcement perceived it as less effective after reading conflicting information (t_{326}=-1.99, p=.048, d=0.22 95% CI [-0.00, 0.44]) ($F_{1,326}$=5.45, p=.020, η_p^2=0.02). Participants thought the vaccine was more effective after the announcement than after reading conflicting information ($F_{1,326}$=232.63, p<.001, η_p^2=0.42) and there was no overall significant difference between those receiving the certain and uncertain announcement ($F_{1,326}$=1, p=.318, η_p^2<0.01).

Government. The certain announcement led to a greater decline in trust and confidence in the government representative after exposure to conflicting information (see Figure 3). Both groups were equally trusting of the government representative after the announcement (t_{326}=-0.54, p=.957, d=0.01 95% CI [-0.21, 0.22]), whereas those who received the certain announcement were less trusting after reading conflicting information (t_{326}=-3.04, p=.003, d=0.34 95% CI [0.12, 0.55]) ($F_{1,326}$=9.54, p=.002, η_p^2=0.03). This interaction means that those who received the certain announcement experienced a greater reduction in trust. Participants had more trust in the government representative after their announcement than after reading conflicting information ($F_{1,326}$=187.12, p<.001, η_p^2=0.37) and there was no overall significant difference between those receiving the certain and uncertain announcement ($F_{1,326}$=2.70, p=.101, η_p^2=0.01).

This was also the case for confidence (see Figure 3). Both groups were equally confident in the government representative after the announcement (t_{326}=0.79, p=.914, d=0.01 95% CI [-

For peer review only - http://bmjopen.bmj.com/site/about/guidelines.xhtml

0.23, 0.21]), whereas those who received the certain announcement were less confident after reading conflicting information (t_{326}=-3.45, p=.001, d=0.38 95% CI [0.16, 0.60]) ($F_{1,326}$=12.08, p=.001, η_p^2=0.04). This means that those who received the certain announcement experienced a greater reduction in confidence. Participants were more confident in the government representative after their announcement than after reading conflicting information ($F_{1,326}$=170.61, p<.001, η_p^2=0.34) and there was no overall significant difference between those receiving the certain and uncertain announcement ($F_{1,326}$=3.13, p=.078, η_p^2=0.01).

Predictors of vaccination intention. In a previous study on communicating uncertainty about vaccines during a pandemic, perceived vaccine effectiveness mediated the relationship between communicated uncertainty and vaccination intention but trust in the government representative did not [18]. We explored whether this was the case here using the PROCESS macro for SPSS [28] (see Figure 4). Both trust in the government representative (b=0.09, 95% CI [0.02,0.18]) and perceived effectiveness (b=0.14, 95% CI [0.003,0.29]) mediated the relationship between announcement certainty and vaccination intention. Participants who received the uncertain announcement were more likely to want to get vaccinated, both because they had higher trust in the government representative and because they perceived the vaccine as more effective after receiving conflicting information. Both of these mechanisms contribute to the effect of uncertainty communication on vaccination intention. Trust may not explain the effect of uncertainty communication on vaccination intention when the announcement is made [18], but it does here after participants are exposed to conflicting information.

Emotions. Although the pattern of findings on emotions is similar, the differences between those receiving the certain and uncertain announcement were less clear, perhaps due to the hypothetical nature of the study. The certain announcement led to a greater increase in avoidance emotions after exposure to conflicting information (see Figure 5). Participants were less worried after the announcement than after reading conflicting information ($F_{1,326}$=60.50, p<.001, η_p^2=0.16), which was qualified by an interaction with the certainty of the announcement ($F_{1,326}$=4.86, p=.028, η_p^2=0.02). Those who received the certain announcement experienced a greater increase in worry than those who received the uncertain announcement, although there was no statistical difference between each group after receiving the announcement (t_{326}=-0.97, p=.332, d=0.11 95% CI [-0.11, 0.32]) or reading the conflicting information (t_{326}=0.51, p=.614, d=0.06 95% CI [-0.16, 0.27]). There was no overall significant difference between those receiving the certain and uncertain announcement ($F_{1,326}$=0.05, p=.819, η_p^2<0.01).

For peer review only - http://bmjopen.bmj.com/site/about/guidelines.xhtml

Participants were less uncertain after the announcement than after reading conflicting information ($F_{1,326}$=19.35, p<.001, η_p^2=0.06), which was qualified by an interaction with the certainty of the announcement ($F_{1,326}$=9.27, p=.003, η_p^2=0.03). Those who received the certain announcement experienced a greater increase in uncertainty than those who received the uncertain announcement, although there was no statistical difference between each group after receiving the announcement (t_{326}=-1.70, p=.091, d=0.19 95% CI [-0.03, 0.40]) or reading the conflicting information (t_{326}=0.74, p=.462, d=0.08 95% CI [-0.14, 0.30]). There was no overall significant difference between those receiving the certain and uncertain announcement ($F_{1,326}$=0.24, p=.628, η_p^2<0.01).

The certain announcement did not lead to a greater decrease in approach emotions after conflicting information (see Figure 5). Participants were more excited about the vaccine after the announcement than after reading conflicting information ($F_{1,326}$=127.76, p<.001, η_p^2=0.28) but the interaction with the certainty of the announcement was marginally significant ($F_{1,326}$=1.20, p=.060, η_p^2=0.01). There was no overall significant difference between those receiving the certain and uncertain announcement ($F_{1,326}$=1.05, p=.306, η_p^2<0.01). Participants were more confident about the vaccine after the announcement than after reading conflicting information ($F_{1,326}$=126.09, p<.001, η_p^2=0.28) but the interaction with the certainty of the announcement was not significant ($F_{1,326}$=2.16, p=.142, η_p^2=0.01). There was no overall difference between those receiving the certain and uncertain announcement ($F_{1,326}$=1.41, p=.235, η_p^2<0.01).

Discussion

Communicating uncertainties had protective effects against new conflicting information. Participants showed a greater reduction in vaccination intention after receiving information which conflicted with communications delivered with certainty, as opposed to communications which acknowledged uncertainties. This was accompanied by a greater reduction in trust in the communicator and perceived vaccine effectiveness, which both affected vaccination intention. Participants also experienced a greater increase in avoidance emotions (worry and uncertainty) following information which conflicted with certain as opposed to uncertain communications. There was no decline in approach emotions, although they were low to begin with.

At the time of the vaccine announcement, we do not find clear evidence that those who received uncertain communications are less likely to get vaccinated. This contrasts with previous findings, although communications in those studies expressed greater uncertainty than

For peer review only - http://bmjopen.bmj.com/site/about/guidelines.xhtml

here [18]. While most of the previous literature indicates that communicating uncertainty has damaging effects [3], our findings are an example of the kinds of contexts in which those effects might be weaker, i.e. when uncertainty is particularly salient. Patients might not expect scientific uncertainty generally [14], but people have been exposed to it during COVID-19 and may therefore expect it and want it communicated [19].

Once people receive information which conflicts with the vaccine announcement, there are differences between those exposed to the certain and uncertain announcement. The government representative who delivered the announcement appears more trustworthy to those who were exposed to uncertainty. Those who received the certain announcement now perceive the vaccine as less effective, although the difference with vaccination intention is less clear. Having said that, those who experience a strong decline in trust and perceived vaccine effectiveness following the certain announcement also experience a strong decline in vaccination intention, making it weaker compared to those who received the uncertain announcement. Although communicating with certainty about vaccines is more damaging for trust in communicators than for vaccination intention, as findings in the financial domain suggested [22], the effects on vaccination intention remain a problem.

Limitations

These findings highlight the benefits of communicating uncertainties in health, but they are only a starting point and should be interpreted with caution. This study focused on uncertainties relating to vaccine effectiveness, but there are many other uncertainties relating to vaccines during a novel pandemic worth exploring. Risks of side effects, including those not detectable in rapid trials, are particularly important to the public when making vaccination decisions [29]. Many are motivated to get vaccinated to reduce the spread of the virus and lift restrictions, but whether the vaccination programme can do so is not necessarily known from the outset [30]. We investigated only the influence of government communications on vaccination intention, but there are many other sources of influence, such as medical professionals, friends and family and social media [31]. In addition, we only exposed participants to one instance of conflicting information, whereas there might be more throughout a pandemic. Vaccination intention and trust are likely to evolve over time and may be more impacted by repeated exposures.

Given the hypothetical nature of the study, caution is warranted when applying findings. We used a hypothetical delay between the vaccine announcement and receiving conflicting information. This makes generalization to real instances more difficult, given that time delays

For peer review only - http://bmjopen.bmj.com/site/about/guidelines.xhtml

increase the likelihood that people forget the information they receive and therefore do not interpret new information as conflicting with it. Having said that, government communications and new information are likely to be highly mediatized and conflicts made salient during a crisis like COVID-19 [1]. In addition, we used a real pandemic situation where participants had prior knowledge and relevant experiences. They are likely to have been more engaged and invested than in completely hypothetical studies.

It would be valuable to know how well these findings generalize beyond a pandemic context in the UK. It is worth investigating whether our findings generalize to other situations, such as physician-patient interactions where communicating uncertainty seems initially problematic but may have long-term benefits that have not been uncovered yet. Generalizing beyond the UK context would be valuable to inform global communication practices. Given that trust in government is important for vaccine uptake beyond the UK [23], we expect findings would be similar in other countries.

Implications for research and policy

These findings highlight the negative consequences of failing to communicate uncertainties. Although communicating with certainty can initially have benefits, if that certainty is not warranted it can have damaging consequences in the long run. Communicators should consider the quality of the evidence and whether people are likely to be exposed to diverging opinions and conflicting information. Anticipating this by discussing uncertainties could avoid negative consequences further down the line. In highly uncertain contexts, people may not actually be averse to uncertainties being communicated, unlike what previous studies in more certain contexts suggest [3]. More work is needed to establish whether people respond differently to uncertain communications depending on the level of contextual uncertainty.

How should uncertainties be communicated? Previous studies suggest some formats are more effective [12]. We used several ways of communicating uncertainty here, which at present cannot be teased apart. We manipulated the uncertainty of vaccine effectiveness, which was a point estimate in the certain announcement and a range in the uncertain announcement. Ranges may communicate uncertainty but they also increase worry and reduce understanding [11], suggesting that they alone are not sufficient. We accompanied the range by an explanation for the uncertainty, which could have increased understanding of the uncertainty. We included verbal descriptions of uncertainty regarding the broader risks and benefits of vaccination which may have increased perception of uncertainty, perhaps making participants respond less

For peer review only - http://bmjopen.bmj.com/site/about/guidelines.xhtml

negatively to conflicting information later on. Future research should evaluate these methods in isolation to better understand their relative effectiveness.

Who is best placed to communicate these uncertainties? This study does not address this, although the following reflections which could inform future research. People might have different expectations of government compared to medical practitioners given they have particularly low levels of trust in politicians [32]. The effects we find on trust could be due to participants perceiving the government as misleading them into getting vaccinated. People might have different expectations of medical practitioners, including certainty in their communications, thereby reacting negatively to uncertainty. Uncertainty could perhaps be interpreted as incompetence from medical practitioners but honesty from politicians. This suggests there may be instances where governments are better placed to communicate uncertainty, particularly during a national crisis, which further research should clarify. In doing so, it is also worth investigating whether political persuasion and government popularity affects trust in government communications and vaccine intention.

Conclusion

During a novel pandemic, where evidence is lacking and evolves over time, people often face changing and conflicting information. Under these circumstances, we show that communicating uncertainties attenuates the negative consequences of being faced with conflicting information. Although it comes with challenges, communicating uncertainty can be beneficial for maintaining trust and patient commitment over time. It takes more account of the potential for health care communications to develop active expertise in its recipients, thereby developing shared and resilient understanding [33,34]. Our findings support calls for greater transparency about uncertainty in communications relating to COVID-19 [35,36].

Acknowledgements

We thank Gerd Gigerenzer for comments on the final manuscript.

Author Contributions

The study was conceptualized by EB and AB. EB collected and analyzed the data. EB, AB, SJ and DT contributed to and approved the final manuscript. The corresponding author attests that all listed authors meet authorship criteria and that no others meeting the criteria have been omitted.

For peer review only - http://bmjopen.bmj.com/site/about/guidelines.xhtml

Funding Source

This research has been supported by the Think Forward Initiative (a partnership between ING Bank, Deloitte, Dell Technologies, Amazon Web Services, IBM, and the Center for Economic Policy Research – CEPR). Grant number not applicable. The views and opinions expressed in this paper are solely those of the authors and do not necessarily reflect the official policy or position of the Think Forward Initiative or any of its partners.

Competing interests

All authors declare: support from the Think Forward Initiative for the funding of the submitted work; no financial relationships with any organisations that might have an interest in the submitted work in the previous three years; no other relationships or activities that could appear to have influenced the submitted work.

Data availability

Anonymized data is available on Open Science Framework (https://osf.io/c73px/).

Ethics approval

Ethical approval for this study was obtained from University College London's Research Ethics Committee (approval number: 15313/001). Participants gave informed consent before taking part.

Transparency declaration

The lead author EB affirms that the manuscript is an honest, accurate, and transparent account of the study being reported; that no important aspects of the study have been omitted; and that any discrepancies from the study as planned (and, if relevant, registered) have been explained.

Dissemination

Dissemination to study participants and patient organisations is not applicable given the study is of interest to policy-makers and practitioners more than patients themselves.

For peer review only - http://bmjopen.bmj.com/site/about/guidelines.xhtml

For peer review only

For peer review only - http://bmjopen.bmj.com/site/about/guidelines.xhtml

References

1 Cohen R. Trust in AstraZeneca vaccine is shaken in Europe. New York Times. 2021.https://www.nytimes.com/2021/03/17/world/europe/AstraZeneca-vaccine-trust-Europe.html

2 Harrison M, Han PKJ, Rabin B, *et al.* Communicating uncertainty in cancer prognosis: A review of web-based prognostic tools. *Patient Educ Couns* 2019;**102**:842–9. doi:10.1016/j.pec.2018.12.009

3 Han PKJ, Babrow A, Hillen MA, *et al.* Uncertainty in health care: Towards a more systematic program of research. *Patient Educ Couns* 2019;**102**:1756–66. doi:10.1016/j.pec.2019.06.012

4 Politi MC, Clark MA, Ombao H, *et al.* Communicating uncertainty can lead to less decision satisfaction: A necessary cost of involving patients in shared decision making? *Heal Expect* 2010;**14**:84–91. doi:10.1111/j.1369-7625.2010.00626.x

5 Gordon GH, Joos SK, Byrne J. Physician expressions of uncertainty during patient encounters. *Patient Educ Couns* 2000;**40**:59–65. doi:10.1016/S0738-3991(99)00069-5

6 Portnoy DB, Han PKJ, Ferrer RA, *et al.* Physicians' attitudes about communicating and managing scientific uncertainty differ by perceived ambiguity aversion of their patients. *Heal Expect* 2011;**16**:362–72. doi:10.1111/j.1369-7625.2011.00717.x

7 Bansback N, Bell M, Spooner L, *et al.* Communicating Uncertainty in Benefits and Harms: A Review of Patient Decision Support Interventions. *Patient* 2017;**10**:311–9. doi:10.1007/s40271-016-0210-z

8 Ogden J, Fuks K, Gardner M, *et al.* Doctors expressions of uncertainty and patient confidence. *Patient Educ Couns* 2002;**48**:171–6. doi:10.1016/S0738-3991(02)00020-4

9 Johnson CG, Levenkron JC, Suchman AL, *et al.* Does physician uncertainty affect patient satisfaction? *J Gen Intern Med* 1988;**3**:144–9. doi:10.1007/BF02596120

10 Schapira MM, Nattinger AB, McHorney CA. Frequency or probability? A qualitative study of risk communication formats used in health care. *Med Decis Mak* 2001;**21**:459–67. doi:10.1177/0272989X0102100604

11 Longman T, Turner RM, King M, *et al.* The effects of communicating uncertainty in quantitative health risk estimates. *Patient Educ Couns* 2012;**89**:252–9. doi:10.1016/j.pec.2012.07.010

12 Han PKJ, Klein WMP, Lehman T, *et al.* Communication of uncertainty regarding individualized cancer risk estimates: Effects and influential factors. *Med Decis Mak* 2011;**31**:354–66. doi:10.1177/0272989X10371830

For peer review only - http://bmjopen.bmj.com/site/about/guidelines.xhtml

13 Han PKJ, Klein WMP, Lehman TC, *et al.* Layperson's responses to the communication of uncertainty regarding cancer risk estimates. *Med Decis Mak* 2009;**29**:391–403. doi:10.1177/0272989X08327396

14 Johnson BB, Slovic P. Lay views on uncertainty in environmental health risk assessment. *J Risk Res* 1998;**1**:261–79. doi:10.1080/136698798377042

15 Van Der Bles AM, Van Der Linden S, Freeman ALJ, *et al.* Communicating uncertainty about facts, numbers and science. *R Soc Open Sci* 2019;**6**. doi:10.1098/rsos.181870

16 Wegwarth O, Wagner GG, Gigerenzer G. Can facts trump unconditional trust? Evidence-based information halves the influence of physicians' non-evidence-based cancer screening recommendations. *PLoS One* 2017;**12**:1–12. doi:10.1371/journal.pone.0183024

17 Johnson BB, Slovic P. Presenting Uncertainty in Health Risk Assessment: Initial Studies of Its Effects on Risk Perception and Trust. *Risk Anal* 1995;**15**:485–94. doi:10.1111/j.1539-6924.1995.tb00341.x

18 Han PKJ, Zikmund-Fisher BJ, Duarte CW, *et al.* Communication of Scientific Uncertainty about a Novel Pandemic Health Threat: Ambiguity Aversion and Its Mechanisms. *J Health Commun* 2018;**23**:435–44. doi:10.1080/10810730.2018.1461961

19 Gustafson A, Rice RE. A review of the effects of uncertainty in public science communication. *Public Underst Sci* 2020;**29**:614–33. doi:10.1177/0963662520942122

20 Dieckmann NF, Mauro R, Slovic P. The effects of presenting imprecise probabilities in intelligence forecasts. *Risk Anal* 2010;**30**:987–1001. doi:10.1111/j.1539-6924.2010.01384.x

21 Jenkins SC, Harris AJL, Lark RM. When unlikely outcomes occur: the role of communication format in maintaining communicator credibility. *J Risk Res* 2019;**22**:537–54. doi:10.1080/13669877.2018.1440415

22 Batteux E, Bilovich A, Johnson SGB, *et al.* When precision backfires: The effects of unwarranted precision on consumer loyalty. *Work Pap* 2020.

23 Lazarus J V, Ratzan S, Palayew A, *et al.* Hesitant or not? A global survey of potential acceptance of a COVID-19 vaccine Jeffrey. *MedRxiv* 2020;**preprint**:1–31. doi:10.1038/s41591-020-1124-9

24 [dataset] Batteux E. Communicating medical uncertainty. Open Sci. Framew. 2020.https://osf.io/c73px/

For peer review only - http://bmjopen.bmj.com/site/about/guidelines.xhtml

25 Tuckett D, Nikolic M. The role of conviction and narrative in decision-making under radical uncertainty. *Theory Psychol* 2017;**27**:501–23. doi:10.1177/0959354317713158

26 Wright L, Steptoe A, Fancourt D. What predicts adherence to COVID-19 government guidelines ? Longitudinal analyses of 51 , 000 UK adults . *MedRxiv* 2020.

27 Martin LR, Petrie KJ. Understanding the Dimensions of Anti-Vaccination Attitudes: the Vaccination Attitudes Examination (VAX) Scale. *Ann Behav Med* Published Online First: 2017. doi:10.1007/s12160-017-9888-y

28 Hayes AF. PROCESS: A versatile computational tool for observed variable mediation, moderation, and conditional process modeling. White Pap. 2012. doi:978-1-60918-230-4

29 Paul E, Steptoe A, Fancourt D. Attitudes towards vaccines and intention to vaccinate against COVID-19: Implications for public health communications. *Lancet Reg Heal - Eur* 2020;**1**. doi:10.1016/j.lanepe.2020.100012

30 Peiris M, Leung GM. What can we expect from first-generation COVID-19 vaccines? *Lancet* 2020;**6736**:20–1. doi:10.1016/s0140-6736(20)31976-0

31 Al-Hasan A, Khuntia J, Yim D. Does Seeing What Others Do Through Social Media Influence Vaccine Uptake and Help in the Herd Immunity Through Vaccination? A Cross-Sectional Analysis. *Front Public Heal* 2021;**9**:1–13. doi:10.3389/fpubh.2021.715931

32 Edelman. Edelman Trust Barometer 2020. Published Online First: 2020. doi:https://www.edelman.com/trustbarometer

33 Tuckett D. Explanatory models and conviction narratives. In: Christmas S, Michie S, West R, eds. *Thinking about Behaviour Change: An Interdisciplinary Dialogue*. Silverback Publishing 2015.

34 Tuckett D, Boulton M, Olson C, *et al. Meetings between experts: An approach to sharing ideas in medical consultations.* London and New York: : Tavistock Publications 1985.

35 Blastland M, Freeman ALJ, Van Der Linden S, *et al.* Five rules for evidence communication. *Nature* 2020;**587**. doi:10.1038/d41586-020-03189-1

36 Sunstein C, Altieri E, Kim M, *et al.* Behavioural considerations for acceptance and uptake of COVID-19 vaccines. 2020.

For peer review only - http://bmjopen.bmj.com/site/about/guidelines.xhtml

Figure legends

Figure 1: CONSORT flow diagram depicting the phases of participant recruitment and analysis.

Figure 2: Vaccination intention and perceived vaccine effectiveness before receiving conflicting information (i.e. after the vaccine announcement) and after receiving conflicting information by announcement certainty.

Figure 3: Trust and confidence in the government representative who made the vaccine announcement before receiving conflicting information (i.e. after the vaccine announcement) and after receiving conflicting information by announcement certainty.

Figure 4: Relationship between announcement certainty and vaccination intention after receiving conflicting information mediated by trust in government representative and perceived vaccine effectiveness. * refers to $p<.05$, ** refers to $p<.01$, *** refers to $p<.001$.

Figure 5: Emotions before receiving conflicting information (i.e. after the vaccine announcement) and after receiving conflicting information by announcement certainty.

For peer review only

For peer review only - http://bmjopen.bmj.com/site/about/guidelines.xhtml

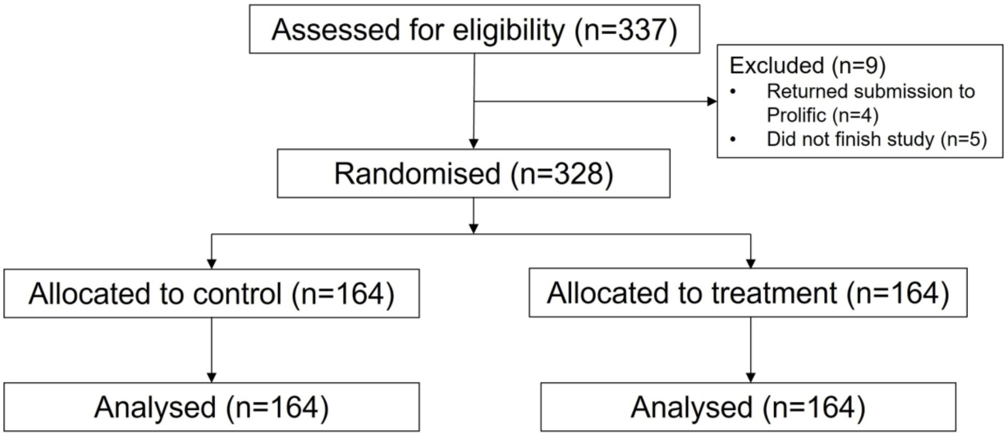

Figure 1: CONSORT flow diagram depicting the phases of participant recruitment and analysis.

212x93mm (300 x 300 DPI)

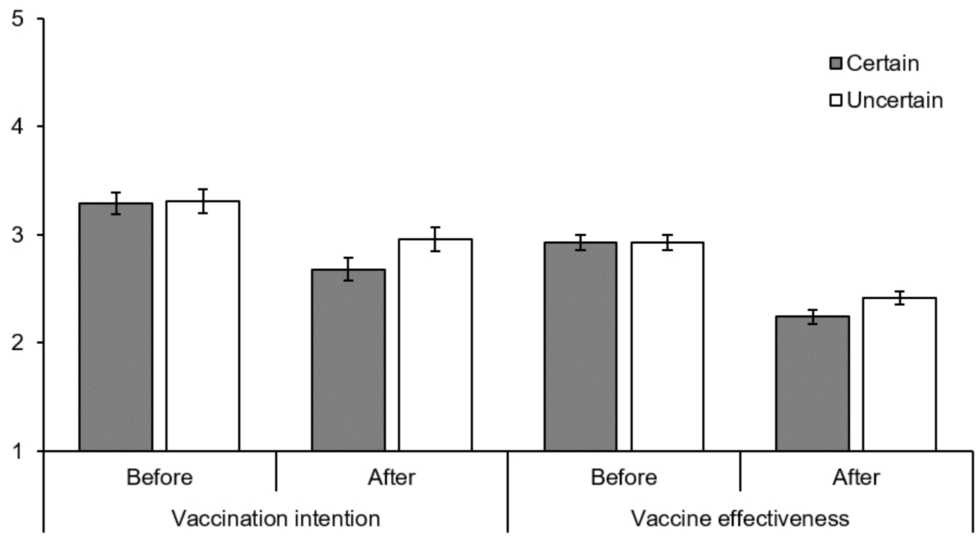

Figure 2: Vaccination intention and perceived vaccine effectiveness before receiving conflicting information (i.e. after the vaccine announcement) and after receiving conflicting information by announcement certainty.

396x218mm (59 x 59 DPI)

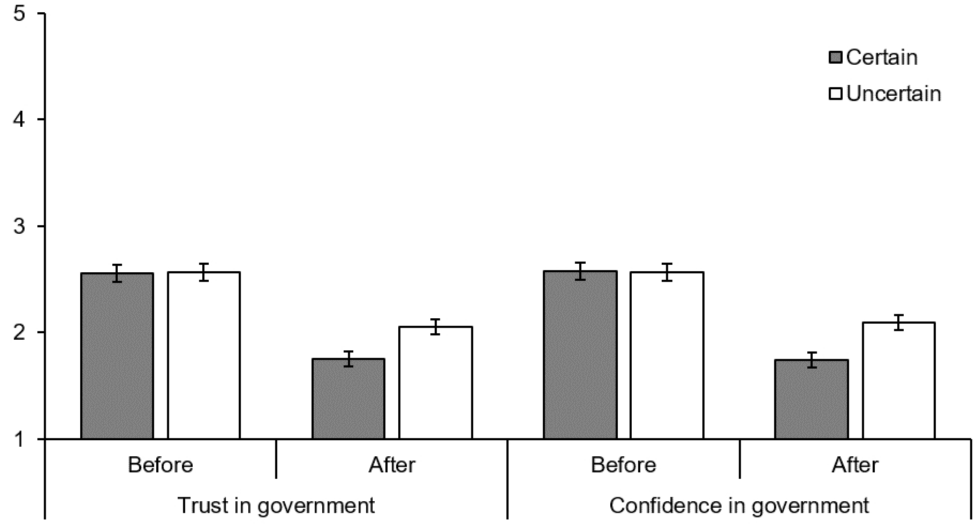

Figure 3: Trust and confidence in the government representative who made the vaccine announcement before receiving conflicting information (i.e. after the vaccine announcement) and after receiving conflicting information by announcement certainty.

401x218mm (59 x 59 DPI)

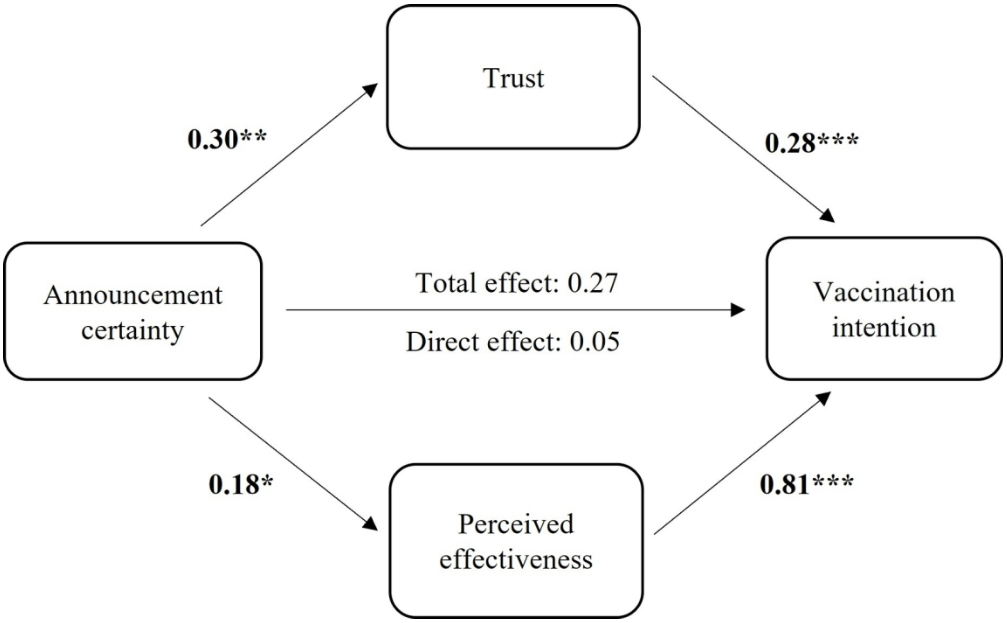

Figure 4: Relationship between announcement certainty and vaccination intention after receiving conflicting information mediated by trust in government representative and perceived vaccine effectiveness. * refers to p<.05, ** refers to p<.01, *** refers to p<.001.

144x88mm (300 x 300 DPI)

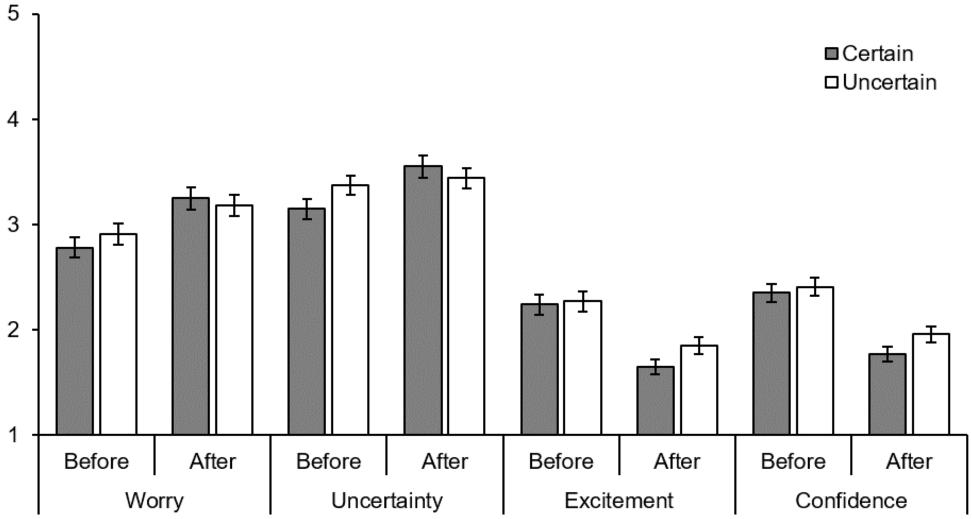

Figure 5: Emotions before receiving conflicting information (i.e. after the vaccine announcement) and after receiving conflicting information by announcement certainty.

404x218mm (59 x 59 DPI)

Appendix: Further analyses on outcome variables

We investigated whether announcement certainty, demographics (age, gender, education) and COVID-19 related beliefs (trust in government, perceived certainty of COVID-19 science, vaccination beliefs) affected our outcome variables (vaccination intention, perceived vaccine effectiveness, trust and confidence in the government official, worry, uncertainty, excitement and confidence). We conducted multiple linear regressions on the differences in each outcome variable between ratings before and after receiving conflicting information (see Table A1). As in our analyses in the main paper, those who received the certain announcement reported the greatest differences between before and after receiving conflicting information. Beliefs towards vaccination also had an effect across most variables, whereby those with more positive beliefs towards vaccination experienced greater differences between before and after receiving conflicting information. This suggests that people who have more positive vaccination beliefs are more likely to be disappointed after receiving conflicting information about vaccine effectiveness. Perhaps this is due to them having greater expectations of vaccine effectiveness and being more surprised once those expectations are not fulfilled.

Table A1: Effects of certainty, demographics and COVID-19 beliefs on differences in outcome before and after conflicting information

	Vaccine		Government		Emotions			
	Vaccination Intention	Effectiveness	Trust	Confidence	Worry	Uncertainty	Excitement	Confidence
Announcement certainty	*B*=-0.27 (0.08)**	*B*=-0.18 (0.08)*	*B*=-0.32 (0.09)***	*B*=-0.39 (0.10)***	*B*=0.23 (0.10)*	*B*=0.35 (0.11)**	*B*=-0.20 (0.09)*	*B*=-0.16 (0.09)
Age	*B*<0.01 (<0.01)	*B*<0.01 (<0.01)	*B*<0.01 (<0.01)	*B*=0.01 (<0.01)	*B*<0.01 (<0.01)	*B*<0.01 (<0.01)	*B*<0.01 (<0.01)	*B*=0.01 (<0.01)**
Gender	*B*=0.08 (0.09)	*B*=0.17 (0.08)*	*B*=0.22 (0.10)*	*B*=0.07 (0.10)	*B*=0.11 (0.10)	*B*=0.03 (0.12)	*B*=0.12 (0.10)	*B*=0.06 (0.10)
Education	*B*<0.01 (0.05)	*B*=-0.03 (0.04)	*B*=-0.05 (0.05)	*B*=0.01 (0.05)	*B*=-0.02 (0.05)	*B*=-0.10 (0.06)	*B*=-0.02 (0.05)	*B*=0.04 (0.05)
Trust in government	*B*=0.08 (0.05)	*B*=0.04 (0.04)	*B*=0.09 (0.05)	*B*=0.14 (0.05)**	*B*<0.01 (0.05)	*B*=-0.03 (0.06)	*B*=0.04 (0.05)	*B*=0.04 (0.05)
Science certainty	*B*=-0.01 (0.01)	*B*<0.01 (0.01)	*B*=0.01 (0.01)	*B*=-0.01 (0.01)	*B*=-0.01 (0.01)	*B*=-0.01 (0.02)	*B*<0.01 (0.01)	*B*=-0.01 (0.01)

For peer review only - http://bmjopen.bmj.com/site/about/guidelines.xhtml

Vaccine beliefs	B=0.01 (0.01)*	B=0.01 (0.01)*	B=0.02 (0.01)***	B=0.03 (0.01)***	B<0.01 (<0.01)	B<0.01 (<0.01)	B=0.02 (0.01)***	B=0.02 (0.01)***
Model	**F=3.01, R^2=6.2%****	**F=2.67, R^2=5.5%***	**F=7.46, R^2=14%*****	**F=8.66, R^2=15.9%*****	F=0.99, R^2=2.1%	F=1.80, R^2=3.8%	**F=4.36, R^2=8.7%*****	**F=5.69, R^2=9.1%*****

Note: Each outcome variable represents the difference in rating before and after receiving conflicting information. Predictor variables are announcement certainty (1=certain, 2=uncertain), age, gender (1=male, 2=female, 3=non-binary), education (1=GCSE or equivalent, 2=A-level or equivalent, 3=undergraduate degree, 4=postgraduate degree), trust in the UK government (scores range from 1-5), beliefs about the certainty of COVID-19 related science (scores range from 3-21), positive beliefs towards vaccination (scores range from 12-60). * refers to $p<.05$, ** $p<.01$, *** $p<.001$.

For peer review only

CONSORT 2010 checklist of information to include when reporting a randomised trial*

Section/Topic	Item No	Checklist item	Reported on page No
**Title and abstract**			
	1a	Identification as a randomised trial in the title	1
	1b	Structured summary of trial design, methods, results, and conclusions (for specific guidance see CONSORT for abstracts)	2
**Introduction**			
Background and objectives	2a	Scientific background and explanation of rationale	1-4
	2b	Specific objectives or hypotheses	4-5
**Methods**			
Trial design	3a	Description of trial design (such as parallel, factorial) including allocation ratio	7
	3b	Important changes to methods after trial commencement (such as eligibility criteria), with reasons	NA
Participants	4a	Eligibility criteria for participants	7
	4b	Settings and locations where the data were collected	7
Interventions	5	The interventions for each group with sufficient details to allow replication, including how and when they were actually administered	9
Outcomes	6a	Completely defined pre-specified primary and secondary outcome measures, including how and when they were assessed	9
	6b	Any changes to trial outcomes after the trial commenced, with reasons	NA
Sample size	7a	How sample size was determined	7-8
	7b	When applicable, explanation of any interim analyses and stopping guidelines	NA
Randomisation:			
Sequence generation	8a	Method used to generate the random allocation sequence	7
	8b	Type of randomisation; details of any restriction (such as blocking and block size)	7
Allocation concealment mechanism	9	Mechanism used to implement the random allocation sequence (such as sequentially numbered containers), describing any steps taken to conceal the sequence until interventions were assigned	7
Implementation	10	Who generated the random allocation sequence, who enrolled participants, and who assigned participants to interventions	7
Blinding	11a	If done, who was blinded after assignment to interventions (for example, participants, care providers, those	7

For peer review only - http://bmjopen.bmj.com/site/about/guidelines.xhtml

		assessing outcomes) and how	
	11b	If relevant, description of the similarity of interventions	NA
Statistical methods	12a	Statistical methods used to compare groups for primary and secondary outcomes	9-14
	12b	Methods for additional analyses, such as subgroup analyses and adjusted analyses	9-14
**Results**			
Participant flow (a diagram is strongly recommended)	13a	For each group, the numbers of participants who were randomly assigned, received intended treatment, and were analysed for the primary outcome	7
	13b	For each group, losses and exclusions after randomisation, together with reasons	NA
Recruitment	14a	Dates defining the periods of recruitment and follow-up	7
	14b	Why the trial ended or was stopped	7
Baseline data	15	A table showing baseline demographic and clinical characteristics for each group	8
Numbers analysed	16	For each group, number of participants (denominator) included in each analysis and whether the analysis was by original assigned groups	9
Outcomes and estimation	17a	For each primary and secondary outcome, results for each group, and the estimated effect size and its precision (such as 95% confidence interval)	9-14
	17b	For binary outcomes, presentation of both absolute and relative effect sizes is recommended	NA
Ancillary analyses	18	Results of any other analyses performed, including subgroup analyses and adjusted analyses, distinguishing pre-specified from exploratory	12
Harms	19	All important harms or unintended effects in each group (for specific guidance see CONSORT for harms)	NA
**Discussion**			
Limitations	20	Trial limitations, addressing sources of potential bias, imprecision, and, if relevant, multiplicity of analyses	15-16
Generalisability	21	Generalisability (external validity, applicability) of the trial findings	15-16
Interpretation	22	Interpretation consistent with results, balancing benefits and harms, and considering other relevant evidence	15
**Other information**			
Registration	23	Registration number and name of trial registry	6
Protocol	24	Where the full trial protocol can be accessed, if available	6
Funding	25	Sources of funding and other support (such as supply of drugs), role of funders	18

*We strongly recommend reading this statement in conjunction with the CONSORT 2010 Explanation and Elaboration for important clarifications on all the items. If relevant, we also recommend reading CONSORT extensions for cluster randomised trials, non-inferiority and equivalence trials, non-pharmacological treatments, herbal interventions, and pragmatic trials. Additional extensions are forthcoming: for those and for up to date references relevant to this checklist, see www.consort-statement.org.

For peer review only

For peer review only - http://bmjopen.bmj.com/site/about/guidelines.xhtml

BMJ Open

The negative consequences of failing to communicate uncertainties during a pandemic: An online randomized controlled trial on COVID-19 vaccines

Journal:	*BMJ Open*
Manuscript ID	bmjopen-2021-051352.R2
Article Type:	Original research
Date Submitted by the Author:	25-Jul-2022
Complete List of Authors:	Batteux, Eleonore; University College London<br>Bilovich, Avri; University College London<br>Johnson, Samuel; University of Warwick<br>Tuckett, David; University College London
**Primary Subject Heading**:	Public health
Secondary Subject Heading:	Health policy
Keywords:	COVID-19, PUBLIC HEALTH, MEDICAL EDUCATION & TRAINING, Health policy < HEALTH SERVICES ADMINISTRATION & MANAGEMENT

SCHOLARONE™
Manuscripts

I, the Submitting Author has the right to grant and does grant on behalf of all authors of the Work (as defined in the below author licence), an exclusive licence and/or a non-exclusive licence for contributions from authors who are: i) UK Crown employees; ii) where BMJ has agreed a CC-BY licence shall apply, and/or iii) in accordance with the terms applicable for US Federal Government officers or employees acting as part of their official duties; on a worldwide, perpetual, irrevocable, royalty-free basis to BMJ Publishing Group Ltd ("BMJ") its licensees and where the relevant Journal is co-owned by BMJ to the co-owners of the Journal, to publish the Work in this journal and any other BMJ products and to exploit all rights, as set out in our licence.

The Submitting Author accepts and understands that any supply made under these terms is made by BMJ to the Submitting Author unless you are acting as an employee on behalf of your employer or a postgraduate student of an affiliated institution which is paying any applicable article publishing charge ("APC") for Open Access articles. Where the Submitting Author wishes to make the Work available on an Open Access basis (and intends to pay the relevant APC), the terms of reuse of such Open Access shall be governed by a Creative Commons licence – details of these licences and which Creative Commons licence will apply to this Work are set out in our licence referred to above.

Other than as permitted in any relevant BMJ Author's Self Archiving Policies, I confirm this Work has not been accepted for publication elsewhere, is not being considered for publication elsewhere and does not duplicate material already published. I confirm all authors consent to publication of this Work and authorise the granting of this licence.

For peer review only

The negative consequences of failing to communicate uncertainties during a pandemic: An online randomized controlled trial on COVID-19 vaccines

Eleonore Batteux[1], Avri Bilovich[1], Samuel G. B. Johnson[2], David Tuckett[1],
[1]University College London, [2]University of Warwick

Corresponding author: David Tuckett, Division of Psychology and Language Sciences, University College London, 1-19 Torrington Place, London, WC1E 7HB, UK, d.tuckett@ucl.ac.uk

Word count: 4,394 words

For peer review only

Abstract

Objective. To examine the impact of the government communicating uncertainties relating to COVID-19 vaccine effectiveness on vaccination intention and trust after people are exposed to conflicting information.

Design. Experimental design where participants were randomly allocated to one of two groups.

Setting. Online.

Participants. 328 adults from a UK research panel.

Intervention. Participants received either certain or uncertain communications from a government representative about COVID-19 vaccine effectiveness, before receiving conflicting information about effectiveness.

Main outcome measures. Vaccination intention and trust in government.

Results. Compared to those who received the uncertain announcement from the government, participants who received the certain announcement reported a greater loss of vaccination intention (d=0.34, 95% CI [0.12, 0.56], p=.002) and trust (d=0.34, 95% CI [0.12, 0.56], p=.002) after receiving conflicting information.

Conclusions. Communicating with certainty about COVID-19 vaccines reduces vaccination intention and trust if conflicting information arises, whereas communicating uncertainties can protect people from the negative impact of exposure to conflicting information. There are likely to be other factors affecting vaccine intentions which we do not account for in this study.

Trial registration number. Open Science Framework: https://osf.io/c73px/

Keywords: uncertainty; health communication; trust; vaccine uptake; COVID-19

For peer review only - http://bmjopen.bmj.com/site/about/guidelines.xhtml

Strengths and limitations of this study

- This study provides experimental evidence of the benefits of communicating with uncertainty rather than certainty during a pandemic.
- Participants were randomly allocated to receive either certain or uncertain hypothetical communications about COVID-19 vaccines.
- Vaccination uptake was measured using a single-item measure of intention.

Introduction

No decision in healthcare comes without a degree of uncertainty. When recommending a treatment, a medical professional generally knows its effectiveness and possible side effects, along with their associated probabilities, i.e. *risks*. They may also be aware there is uncertainty surrounding that probability estimate, sometimes called *ambiguity* or *radical uncertainty*. This kind of uncertainty is particularly salient in a pandemic, where the precise outcomes of treatments and policies cannot be known. Earlier on in the COVID-19 vaccine roll out, research was still underway to confirm vaccines' effectiveness and risks. Accounts of damaging side effects, such as thrombosis following the AstraZeneca vaccine, severely damaged trust [1]. Today, there remain uncertainties about the effectiveness of vaccines against new variants.

Despite the prevalence of uncertainty, there is a lack of consensus on how best to communicate it [2]. A first step has been to investigate how patients respond to communications of uncertainty, which has largely uncovered negative impacts and led to interrogations on how best to communicate it (if at all) [3]. We take a different approach in this paper, where we investigate the negative consequences of *failing* to communicate uncertainties. Are there times where, however difficult it may be to communicate uncertainties, doing so is better than hiding them? Does failing to communicate uncertainties backfire if people find out they exist and are exposed to conflicting information? We explore these questions by investigating how people respond to conflicting COVID-19 vaccine communications.

Communicating uncertainty in health

In this paper, we distinguish risk or probabilistic uncertainty (e.g. 20% chance of benefit from treatment) from uncertainty, or what can also be referred to as ambiguity. Uncertainty can take various forms: imprecision (e.g. 10-30% chance of benefit from treatment), conflict (e.g. experts disagreeing), lack of information (e.g. insufficient evidence) [3]. All three are present during a pandemic like COVID-19, so we consider them together here.

For peer review only - http://bmjopen.bmj.com/site/about/guidelines.xhtml

Uncertainty is communicated to varying degrees across healthcare. Physicians mention some form of uncertainty in most of their patient encounters, although this tends to be in vague terms (e.g. 'There is a chance it will/won't work') [4] [5]. However, physicians are less likely to report that they would communicate uncertainty if they believe patients will have negative reactions to it, which tends to be the case [6]. Interventions designed to communicate information to patients often include quantitative risk estimates, but mentioning uncertainty tends to be the exception [2,7]. When mentioned, it is usually verbally (e.g. "about" or "up to"). This highlights the lack of consensus for how and when to communicate uncertainty in health.

This is not surprising given that uncertainty can have negative effects on patients, for both significant (e.g. cancer, [4]) and more minor (e.g. acne [8]) illnesses. Verbal expressions of uncertainty by doctors can lower patient confidence [8] and satisfaction [4,9]. Numerical expressions of uncertainty (e.g. ranges) can reduce trust and credibility [10,11] and increase perceptions of risk and worry, although less so when communicated visually compared to textually [11–13]. This could be because people generally think science can provide certainty [14] and therefore interpret expressions of uncertainty as signs of incompetence rather than an inevitable feature of science. Explaining why there is uncertainty might help to mitigate misunderstandings, which has been recommended when communicating uncertainty in general [15]. In addition, providing numerical information about risks and benefits makes patients less likely to overturn their decision in the face of conflicting information [16].

We focus on the effects of communicating uncertainty in public health, which present notable differences. Discussing uncertainty around numerical risk estimates may decrease perceived competence but also increase perceived honesty [14,17]. Although people report preferring to see precision in communications, they would rather uncertainties be disclosed if they exist [14]. This suggests that if people are aware that uncertainties exist, they may be suspicious of communications which do not mention them. Nonetheless, a previous study on vaccine communications during a hypothetical novel pandemic found that uncertain communications led to lower vaccination intention and lower trust in the communicator [18]. However, this may be because the communications were verbal and highly uncertain (e.g. "we are not sure exactly how effective it will be"). There is more precise information in the context of COVID-19, despite prevailing uncertainties.

What if uncertainties are not communicated?

When uncertainties do exist, can ignoring them backfire? The literature indicates there are advantages to not communicating uncertainties, but it does not address the consequences

For peer review only - http://bmjopen.bmj.com/site/about/guidelines.xhtml

once people are confronted with information which conflicts with what they were communicated. There are many instances where this applies. A vaccine might be 70% effective against infection, but that does not mean the vaccinated are certain they will not get infected. In contexts where evidence is lacking, new evidence can arise which invalidates previous communications. Although disclosing uncertainties might have negative effects initially, over time it could protect against the consequences of people experiencing undesirable outcomes or conflicting information, which is damaging in science communication [19].

In other contexts, communicating uncertainty can be beneficial in the long term. In an intelligence context, when people are told a terrorist attack occurred and shown the forecasts, they find forecasters who communicated uncertainty more credible and less worthy of blame [20]. In a geological context, there is no evidence of a difference between certain and uncertain forecasts in terms of perceived correctness and loss of credibility after unlikely events occur [21]. In a financial investment context, when forecasts of future returns turn out to be incorrect, forecasters who did not acknowledge uncertainty were perceived as less trustworthy [22]. Interestingly, this did not lead investors to pull out of their investment, showing that they blame the forecaster for incorrect forecasts but not the object of the forecast. It is worth investigating whether this applies to a medical context, i.e. whether failing to communicate uncertainties has worse consequences for confidence in the communicator than in the object of the communication (e.g. a treatment or vaccine).

The present research

We examine how uncertain communications affect trust and vaccination intention over time. Specifically, we test whether communicating uncertainty about COVID-19 vaccines limits any loss of trust and vaccination intention after people receive conflicting information about their effectiveness. We focus on COVID-19 given the need to maximize vaccine uptake, where low trust has been linked to vaccine hesitancy [23]. In addition, COVID-19 provides a real pandemic context that participants can relate to and have knowledge of. Our hypotheses were preregistered on the Open Science Framework [24] and are as follows.

Hypothesis 1: We expect people are less favorable to vaccination after receiving uncertain compared to certain communications. The first main outcome variable is vaccination intention, which we expect to be lower following uncertain communications, as found in a previous study [18]. We investigate whether this is accompanied by lower perceptions of vaccine effectiveness [18], stronger avoidance emotions (e.g. worry) and weaker approach

For peer review only - http://bmjopen.bmj.com/site/about/guidelines.xhtml

emotions (e.g. excitement). Indeed, emotions are crucial to decision-making in contexts of uncertainty [25]. The second main outcome variable is trust in communicators, which is crucial to both vaccine uptake and compliance to guidelines during a pandemic [23,26]. Previous studies suggest trust should be lower when uncertainty is communicated [10,18].

Hypothesis 2: Once people receive information which conflicts with earlier communications, we expect those who initially received certain communications to experience more negative effects compared to those who received uncertain communications. We posit that communicating uncertainty makes people more likely to expect information to change over time and therefore less affected when confronted to conflicting information. On the other hand, communicating with unwarranted certainty may be perceived as intentionally misleading. We expect to see greater reductions in vaccination intention in those receiving certain communications.

We conducted a study in November 2020, before COVID-19 effectiveness rates were widely communicated. We presented participants from the general UK population with a hypothetical vaccine announcement containing information about the vaccine's effectiveness, which either conveyed certainty or uncertainty. Participants were then given new information about vaccine effectiveness, which conflicts with the earlier announcement. We compare participants' vaccination intention, trust, perceived vaccine effectiveness and affective reactions after receiving the announcement to after receiving conflicting information.

Method

Trial design. Communication certainty (1-certain, 2-uncertain) was manipulated between-subjects.

Participants. 328 participants residing in the UK were recruited using Prolific, an online participant recruitment platform (https://www.prolific.co/) (see Figure 1). A sample of 328 was required to find a small effect (d=0.20) for Hypotheses 2a-e with a mixed model ANOVA with high power (>.95) and alpha level (<.05). This sample size also allows enough power to test Hypothesis 1 in accordance with existing findings. Participants were compensated for their time at a rate of £7.50 per hour. They were asked demographic questions (age, gender, level of education). They were then asked questions about COVID-19; firstly, how much trust they currently have in the government's handling of the COVID-19 crisis on a 5-point scale (1-not at all, 5-a great deal). Secondly, how reliable, precise and consistent they perceive the science relating to COVID-19 on a 7-point scale (1-reliable/precise/consistent, 7-unreliable/imprecise/inconsistent). These were added to provide an overall score on their

For peer review only - http://bmjopen.bmj.com/site/about/guidelines.xhtml

perception of the certainty of COVID-19 science. Finally, participants completed the Vaccination Attitudes Examination scale which provides an overall score of favorability to vaccination [27] on a 5-point scale (1-strongly disagree, 5-strongly agree). Participant characteristics can be found in Table 1.

Patient and public involvement. The public was involved in the development of the communications used in the study. We conducted an online pilot study with 50 UK participants to check that the communications about vaccine effectiveness were understandable and believable, with the opportunity for participants to provide feedback.

Table 1: Participant characteristics

Demographics		
	Age	*M*=35.09 (*SD*=11.36)
	Gender	28% Male
		71% Female
		1% Non-binary
	Education	11% GCSE or equivalent
		23.5% A-level or equivalent
		45% Undergraduate degree
		20% Postgraduate degree
Beliefs		
	Trust in gov	*M*=2.13 (*SD*=0.99)
	Science certainty	*M*=11.47 (*SD*=4.10)
	Vaccinations	*M*=39.97 (*SD*=10.02)

Note: Trust in government can range from 1-5, science certainty from 3-21, and vaccination attitudes from 12-60 (with higher figures indicating more favorable attitudes to vaccination).

Interventions. Participants were reminded they are in the middle of the COVID-19 pandemic and told to imagine they hear a public health government representative make a vaccine announcement on the news. This announcement states that a vaccine has passed the necessary checks and will soon be available. For those in the certain condition the representative says: "I can confirm that the vaccine is 60% effective. This means that, although the vaccine might not work for everyone, there is a very good chance that it will work for you. This vaccine will significantly drive down the infection rate and we will be able to remove the restrictive measures we put in place to combat the virus." In the uncertain condition the

For peer review only - http://bmjopen.bmj.com/site/about/guidelines.xhtml

representative says: “The vaccine is between 50 and 70% effective. The reason I can't give a more precise estimate is because the data we have doesn't allow that. There might be some things we don't know yet about the vaccine, but this is the best available option. Although it might not work for everyone, there is a chance it will work for you. This vaccine will hopefully drive down the infection rate and we may be able to remove the restrictive measures we put in place to combat the virus.” Then, all participants are told: “a week later, the vaccine is available and you can book an appointment with your local GP practice. Before deciding whether to get it, you want to read the research on the vaccine's effectiveness. You find the latest international piece of research which is deemed to have the most reliable data. This tells you that the vaccine is actually nearer to 40% effective.”

Outcomes. Measures were taken after participants received the initial announcement and after they read the additional research about the vaccine’s effectiveness. Participants were asked how much confidence and trust they have in the government representative, how effective they think the vaccine is, how they feel about getting the vaccine (excited, confident, worried, uncertain) on 5-point scales (1-not at all, 5-a great deal) and how likely they are to get the vaccine on a 5-point scale (1-definitely not, 5-definitely yes).

Randomisation and blinding. Participants were randomly allocated to the certain or uncertain communication condition via the Qualtrics survey platform randomization function and were blind to the condition they were allocated to.

Statistical methods. As specified in the preregistered analysis plan, Hypotheses 1 and 2 were tested with mixed model ANOVAs. Announcement certainty (1-certain; 2-uncertain) was a between subjects factor and time point (1-after announcement; 2-after conflicting information) was a within subjects factor. This analysis was conducted for all dependent measures (vaccination intention, effectiveness, trust, confidence, emotions). Outcome assessors were not blind to the treatment group participants were allocated to.

Results

The findings are broadly consistent across measures of vaccination intention, vaccine effectiveness, trust and confidence in government and emotion. They support the hypothesis that conflicting information leads to more negative effects among those who were exposed to certain compared to uncertain communications (Hypothesis 2). However, they do not support the hypothesis that people are initially more favorable to certain compared to uncertain communications (Hypothesis 1). Further analyses with demographics and Covid-19 related beliefs are detailed in the Supplementary File, which broadly do not affect our findings.

For peer review only - http://bmjopen.bmj.com/site/about/guidelines.xhtml

Vaccination. The certain announcement led to a greater decline in vaccination intention following exposure to conflicting information (see Figure 2). There was no difference in vaccination intention between people who received the certain and uncertain announcement after the announcement (t_{326}=-0.12, p=.903, d=0.01 95% CI [-0.20, 0.23]), but there was a marginal difference after reading the conflicting information (t_{326}=-1.804, p=.072, d=0.20 95% CI [0.02, 0.42]) ($F_{1,326}$=9.50, p=.002, η_p^2=0.03). The significant interaction indicates that those who received the certain announcement experienced a greater reduction in vaccination intention than those who received the uncertain announcement. Participants had stronger vaccination intentions after the announcement than after reading conflicting information ($F_{1,326}$=134.47, p<.001, η_p^2=0.29) and there was no overall difference between those receiving the certain and uncertain announcement ($F_{1,326}$=1.02, p=.314, η_p^2<0.01).

The pattern was the same for effectiveness, where the certain announcement led to a greater decline in perceived effectiveness (see Figure 2). After the announcement, perceptions of effectiveness were comparable between those who received the certain and uncertain announcement (t_{326}=0.06, p=.951, d=0.01 95% CI [-0.23, 0.21]), whereas those who received the certain announcement perceived it as less effective after reading conflicting information (t_{326}=-1.99, p=.048, d=0.22 95% CI [-0.00, 0.44]) ($F_{1,326}$=5.45, p=.020, η_p^2=0.02). Participants thought the vaccine was more effective after the announcement than after reading conflicting information ($F_{1,326}$=232.63, p<.001, η_p^2=0.42) and there was no overall significant difference between those receiving the certain and uncertain announcement ($F_{1,326}$=1, p=.318, η_p^2<0.01).

Government. The certain announcement led to a greater decline in trust and confidence in the government representative after exposure to conflicting information (see Figure 3). Both groups were equally trusting of the government representative after the announcement (t_{326}=-0.54, p=.957, d=0.01 95% CI [-0.21, 0.22]), whereas those who received the certain announcement were less trusting after reading conflicting information (t_{326}=-3.04, p=.003, d=0.34 95% CI [0.12, 0.55]) ($F_{1,326}$=9.54, p=.002, η_p^2=0.03). This interaction means that those who received the certain announcement experienced a greater reduction in trust. Participants had more trust in the government representative after their announcement than after reading conflicting information ($F_{1,326}$=187.12, p<.001, η_p^2=0.37) and there was no overall significant difference between those receiving the certain and uncertain announcement ($F_{1,326}$=2.70, p=.101, η_p^2=0.01).

This was also the case for confidence (see Figure 3). Both groups were equally confident in the government representative after the announcement (t_{326}=0.79, p=.914, d=0.01 95% CI [-

For peer review only - http://bmjopen.bmj.com/site/about/guidelines.xhtml

0.23, 0.21]), whereas those who received the certain announcement were less confident after reading conflicting information (t_{326}=-3.45, p=.001, d=0.38 95% CI [0.16, 0.60]) ($F_{1,326}$=12.08, p=.001, η_p^2=0.04). This means that those who received the certain announcement experienced a greater reduction in confidence. Participants were more confident in the government representative after their announcement than after reading conflicting information ($F_{1,326}$=170.61, p<.001, η_p^2=0.34) and there was no overall significant difference between those receiving the certain and uncertain announcement ($F_{1,326}$=3.13, p=.078, η_p^2=0.01).

Predictors of vaccination intention. In a previous study on communicating uncertainty about vaccines during a pandemic, perceived vaccine effectiveness mediated the relationship between communicated uncertainty and vaccination intention but trust in the government representative did not [18]. We explored whether this was the case here using the PROCESS macro for SPSS [28] (see Figure 4). Both trust in the government representative (b=0.09, 95% CI [0.02,0.18]) and perceived effectiveness (b=0.14, 95% CI [0.003,0.29]) mediated the relationship between announcement certainty and vaccination intention. Participants who received the uncertain announcement were more likely to want to get vaccinated, both because they had higher trust in the government representative and because they perceived the vaccine as more effective after receiving conflicting information. Both of these mechanisms contribute to the effect of uncertainty communication on vaccination intention. Trust may not explain the effect of uncertainty communication on vaccination intention when the announcement is made [18], but it does here after participants are exposed to conflicting information.

Emotions. Although the pattern of findings on emotions is similar, the differences between those receiving the certain and uncertain announcement were less clear, perhaps due to the hypothetical nature of the study. The certain announcement led to a greater increase in avoidance emotions after exposure to conflicting information (see Figure 5). Participants were less worried after the announcement than after reading conflicting information ($F_{1,326}$=60.50, p<.001, η_p^2=0.16), which was qualified by an interaction with the certainty of the announcement ($F_{1,326}$=4.86, p=.028, η_p^2=0.02). Those who received the certain announcement experienced a greater increase in worry than those who received the uncertain announcement, although there was no statistical difference between each group after receiving the announcement (t_{326}=-0.97, p=.332, d=0.11 95% CI [-0.11, 0.32]) or reading the conflicting information (t_{326}=0.51, p=.614, d=0.06 95% CI [-0.16, 0.27]). There was no overall significant difference between those receiving the certain and uncertain announcement ($F_{1,326}$=0.05, p=.819, η_p^2<0.01).

For peer review only - http://bmjopen.bmj.com/site/about/guidelines.xhtml

Participants were less uncertain after the announcement than after reading conflicting information ($F_{1,326}$=19.35, p<.001, η_p^2=0.06), which was qualified by an interaction with the certainty of the announcement ($F_{1,326}$=9.27, p=.003, η_p^2=0.03). Those who received the certain announcement experienced a greater increase in uncertainty than those who received the uncertain announcement, although there was no statistical difference between each group after receiving the announcement (t_{326}=-1.70, p=.091, d=0.19 95% CI [-0.03, 0.40]) or reading the conflicting information (t_{326}=0.74, p=.462, d=0.08 95% CI [-0.14, 0.30]). There was no overall significant difference between those receiving the certain and uncertain announcement ($F_{1,326}$=0.24, p=.628, η_p^2<0.01).

The certain announcement did not lead to a greater decrease in approach emotions after conflicting information (see Figure 5). Participants were more excited about the vaccine after the announcement than after reading conflicting information ($F_{1,326}$=127.76, p<.001, η_p^2=0.28) but the interaction with the certainty of the announcement was marginally significant ($F_{1,326}$=1.20, p=.060, η_p^2=0.01). There was no overall significant difference between those receiving the certain and uncertain announcement ($F_{1,326}$=1.05, p=.306, η_p^2<0.01). Participants were more confident about the vaccine after the announcement than after reading conflicting information ($F_{1,326}$=126.09, p<.001, η_p^2=0.28) but the interaction with the certainty of the announcement was not significant ($F_{1,326}$=2.16, p=.142, η_p^2=0.01). There was no overall difference between those receiving the certain and uncertain announcement ($F_{1,326}$=1.41, p=.235, η_p^2<0.01).

Discussion

Communicating uncertainties had protective effects against new conflicting information. Participants showed a greater reduction in vaccination intention after receiving information which conflicted with communications delivered with certainty, as opposed to communications which acknowledged uncertainties. This was accompanied by a greater reduction in trust in the communicator and perceived vaccine effectiveness, which both affected vaccination intention. Participants also experienced a greater increase in avoidance emotions (worry and uncertainty) following information which conflicted with certain as opposed to uncertain communications. There was no decline in approach emotions, although they were low to begin with.

At the time of the vaccine announcement, we do not find clear evidence that those who received uncertain communications are less likely to get vaccinated. This contrasts with previous findings, although communications in those studies expressed greater uncertainty than

For peer review only - http://bmjopen.bmj.com/site/about/guidelines.xhtml

here [18]. While most of the previous literature indicates that communicating uncertainty has damaging effects [3], our findings are an example of the kinds of contexts in which those effects might be weaker, i.e. when uncertainty is particularly salient. Patients might not expect scientific uncertainty generally [14], but people have been exposed to it during COVID-19 and may therefore expect it and want it communicated [19].

Once people receive information which conflicts with the vaccine announcement, there are differences between those exposed to the certain and uncertain announcement. The government representative who delivered the announcement appears more trustworthy to those who were exposed to uncertainty. Those who received the certain announcement now perceive the vaccine as less effective, although the difference with vaccination intention is less clear. Having said that, those who experience a strong decline in trust and perceived vaccine effectiveness following the certain announcement also experience a strong decline in vaccination intention, making it weaker compared to those who received the uncertain announcement. Although communicating with certainty about vaccines is more damaging for trust in communicators than for vaccination intention, as findings in the financial domain suggested [22], the effects on vaccination intention remain a problem.

Limitations

These findings highlight the benefits of communicating uncertainties in health, but they are only a starting point and should be interpreted with caution. This study focused on uncertainties relating to vaccine effectiveness, but there are many other uncertainties relating to vaccines during a novel pandemic worth exploring. Risks of side effects, including those not detectable in rapid trials, are particularly important to the public when making vaccination decisions [29]. Many are motivated to get vaccinated to reduce the spread of the virus and lift restrictions, but whether the vaccination programme can do so is not necessarily known from the outset [30]. We investigated only the influence of government communications on vaccination intention, but there are many other sources of influence, such as medical professionals, friends and family and social media [31]. In addition, we only exposed participants to one instance of conflicting information, whereas there might be more throughout a pandemic. Vaccination intention and trust are likely to evolve over time and may be more impacted by repeated exposures.

Given the hypothetical nature of the study, caution is warranted when applying findings. We used a hypothetical delay between the vaccine announcement and receiving conflicting information. This makes generalization to real instances more difficult, given that time delays

For peer review only - http://bmjopen.bmj.com/site/about/guidelines.xhtml

increase the likelihood that people forget the information they receive and therefore do not interpret new information as conflicting with it. Having said that, government communications and new information are likely to be highly mediatized and conflicts made salient during a crisis like COVID-19 [1]. In addition, we used a real pandemic situation where participants had prior knowledge and relevant experiences. They are likely to have been more engaged and invested than in completely hypothetical studies.

It is worth noting that we did not ask participants whether they had been previously diagnosed with COVID-19. It is unclear whether it would have affected their vaccine intentions, although unlikely to be a confound here since participants were randomly assigned to the control and treatment conditions. Previously having had COVID-19 could have made participants feel more strongly about having certainty over vaccine effectiveness due to negative experiences, or less strongly since they could now believe they are immune.

It would be valuable to know how well these findings generalize beyond a pandemic context in the UK. It is worth investigating whether our findings generalize to other situations, such as physician-patient interactions where communicating uncertainty seems initially problematic but may have long-term benefits that have not been uncovered yet. Generalizing beyond the UK context would be valuable to inform global communication practices. Given that trust in government is important for vaccine uptake beyond the UK [23], we expect findings would be similar in other countries.

Implications for research and policy

These findings highlight the negative consequences of failing to communicate uncertainties. Although communicating with certainty can initially have benefits, if that certainty is not warranted it can have damaging consequences in the long run. Communicators should consider the quality of the evidence and whether people are likely to be exposed to diverging opinions and conflicting information. Anticipating this by discussing uncertainties could avoid negative consequences further down the line. In highly uncertain contexts, people may not actually be averse to uncertainties being communicated, unlike what previous studies in more certain contexts suggest [3]. More work is needed to establish whether people respond differently to uncertain communications depending on the level of contextual uncertainty.

How should uncertainties be communicated? Previous studies suggest some formats are more effective [12]. We used several ways of communicating uncertainty here, which at present cannot be teased apart. We manipulated the uncertainty of vaccine effectiveness, which was a point estimate in the certain announcement and a range in the uncertain announcement. Ranges

For peer review only - http://bmjopen.bmj.com/site/about/guidelines.xhtml

may communicate uncertainty but they also increase worry and reduce understanding [11], suggesting that they alone are not sufficient. We accompanied the range by an explanation for the uncertainty, which could have increased understanding of the uncertainty. We included verbal descriptions of uncertainty regarding the broader risks and benefits of vaccination which may have increased perception of uncertainty, perhaps making participants respond less negatively to conflicting information later on. Future research should evaluate these methods in isolation to better understand their relative effectiveness.

Who is best placed to communicate these uncertainties? This study does not address this, although the following reflections which could inform future research. People might have different expectations of government compared to medical practitioners given they have particularly low levels of trust in politicians [32]. The effects we find on trust could be due to participants perceiving the government as misleading them into getting vaccinated. People might have different expectations of medical practitioners, including certainty in their communications, thereby reacting negatively to uncertainty. Uncertainty could perhaps be interpreted as incompetence from medical practitioners but honesty from politicians. This suggests there may be instances where governments are better placed to communicate uncertainty, particularly during a national crisis, which further research should clarify. In doing so, it is also worth investigating whether political persuasion and government popularity affects trust in government communications and vaccine intention.

Conclusion

During a novel pandemic, where evidence is lacking and evolves over time, people often face changing and conflicting information. Under these circumstances, we show that the government communicating uncertainties attenuates the negative consequences of being faced with conflicting information. Although it comes with challenges, communicating uncertainty can be beneficial for maintaining trust and patient commitment over time. It takes more account of the potential for health care communications to develop active expertise in its recipients, thereby developing shared and resilient understanding [33,34]. Our findings support calls for greater transparency about uncertainty in communications relating to COVID-19 [35,36].

Acknowledgements

We thank Gerd Gigerenzer for comments on the final manuscript.

For peer review only - http://bmjopen.bmj.com/site/about/guidelines.xhtml

Author Contributions

The study was conceptualized by EB and AB. EB collected and analyzed the data. EB, AB, SJ and DT contributed to and approved the final manuscript. The corresponding author attests that all listed authors meet authorship criteria and that no others meeting the criteria have been omitted.

Funding Source

This research has been supported by the Think Forward Initiative (a partnership between ING Bank, Deloitte, Dell Technologies, Amazon Web Services, IBM, and the Center for Economic Policy Research – CEPR). Grant number not applicable. The views and opinions expressed in this paper are solely those of the authors and do not necessarily reflect the official policy or position of the Think Forward Initiative or any of its partners.

Competing interests

All authors declare: support from the Think Forward Initiative for the funding of the submitted work; no financial relationships with any organisations that might have an interest in the submitted work in the previous three years; no other relationships or activities that could appear to have influenced the submitted work.

Data availability

Anonymized data is available on Open Science Framework (https://osf.io/c73px/).

Ethics approval

Ethical approval for this study was obtained from University College London's Research Ethics Committee (approval number: 15313/001). Participants gave written informed consent online before taking part.

Transparency declaration

The lead author EB affirms that the manuscript is an honest, accurate, and transparent account of the study being reported; that no important aspects of the study have been omitted; and that any discrepancies from the study as planned (and, if relevant, registered) have been explained.

For peer review only - http://bmjopen.bmj.com/site/about/guidelines.xhtml

Dissemination

Dissemination to study participants and patient organisations is not applicable given the study is of interest to policymakers and practitioners more than patients themselves.

For peer review only

For peer review only - http://bmjopen.bmj.com/site/about/guidelines.xhtml

References

1 Cohen R. Trust in AstraZeneca vaccine is shaken in Europe. New York Times. 2021.https://www.nytimes.com/2021/03/17/world/europe/AstraZeneca-vaccine-trust-Europe.html

2 Harrison M, Han PKJ, Rabin B, *et al.* Communicating uncertainty in cancer prognosis: A review of web-based prognostic tools. *Patient Educ Couns* 2019;**102**:842–9. doi:10.1016/j.pec.2018.12.009

3 Han PKJ, Babrow A, Hillen MA, *et al.* Uncertainty in health care: Towards a more systematic program of research. *Patient Educ Couns* 2019;**102**:1756–66. doi:10.1016/j.pec.2019.06.012

4 Politi MC, Clark MA, Ombao H, *et al.* Communicating uncertainty can lead to less decision satisfaction: A necessary cost of involving patients in shared decision making? *Heal Expect* 2010;**14**:84–91. doi:10.1111/j.1369-7625.2010.00626.x

5 Gordon GH, Joos SK, Byrne J. Physician expressions of uncertainty during patient encounters. *Patient Educ Couns* 2000;**40**:59–65. doi:10.1016/S0738-3991(99)00069-5

6 Portnoy DB, Han PKJ, Ferrer RA, *et al.* Physicians' attitudes about communicating and managing scientific uncertainty differ by perceived ambiguity aversion of their patients. *Heal Expect* 2011;**16**:362–72. doi:10.1111/j.1369-7625.2011.00717.x

7 Bansback N, Bell M, Spooner L, *et al.* Communicating Uncertainty in Benefits and Harms: A Review of Patient Decision Support Interventions. *Patient* 2017;**10**:311–9. doi:10.1007/s40271-016-0210-z

8 Ogden J, Fuks K, Gardner M, *et al.* Doctors expressions of uncertainty and patient confidence. *Patient Educ Couns* 2002;**48**:171–6. doi:10.1016/S0738-3991(02)00020-4

9 Johnson CG, Levenkron JC, Suchman AL, *et al.* Does physician uncertainty affect patient satisfaction? *J Gen Intern Med* 1988;**3**:144–9. doi:10.1007/BF02596120

10 Schapira MM, Nattinger AB, McHorney CA. Frequency or probability? A qualitative study of risk communication formats used in health care. *Med Decis Mak* 2001;**21**:459–67. doi:10.1177/0272989X0102100604

11 Longman T, Turner RM, King M, *et al.* The effects of communicating uncertainty in quantitative health risk estimates. *Patient Educ Couns* 2012;**89**:252–9. doi:10.1016/j.pec.2012.07.010

12 Han PKJ, Klein WMP, Lehman T, *et al.* Communication of uncertainty regarding individualized cancer risk estimates: Effects and influential factors. *Med Decis Mak* 2011;**31**:354–66. doi:10.1177/0272989X10371830

For peer review only - http://bmjopen.bmj.com/site/about/guidelines.xhtml

13 Han PKJ, Klein WMP, Lehman TC, *et al.* Layperson's responses to the communication of uncertainty regarding cancer risk estimates. *Med Decis Mak* 2009;**29**:391–403. doi:10.1177/0272989X08327396

14 Johnson BB, Slovic P. Lay views on uncertainty in environmental health risk assessment. *J Risk Res* 1998;**1**:261–79. doi:10.1080/136698798377042

15 Van Der Bles AM, Van Der Linden S, Freeman ALJ, *et al.* Communicating uncertainty about facts, numbers and science. *R Soc Open Sci* 2019;**6**. doi:10.1098/rsos.181870

16 Wegwarth O, Wagner GG, Gigerenzer G. Can facts trump unconditional trust? Evidence-based information halves the influence of physicians' non-evidence-based cancer screening recommendations. *PLoS One* 2017;**12**:1–12. doi:10.1371/journal.pone.0183024

17 Johnson BB, Slovic P. Presenting Uncertainty in Health Risk Assessment: Initial Studies of Its Effects on Risk Perception and Trust. *Risk Anal* 1995;**15**:485–94. doi:10.1111/j.1539-6924.1995.tb00341.x

18 Han PKJ, Zikmund-Fisher BJ, Duarte CW, *et al.* Communication of Scientific Uncertainty about a Novel Pandemic Health Threat: Ambiguity Aversion and Its Mechanisms. *J Health Commun* 2018;**23**:435–44. doi:10.1080/10810730.2018.1461961

19 Gustafson A, Rice RE. A review of the effects of uncertainty in public science communication. *Public Underst Sci* 2020;**29**:614–33. doi:10.1177/0963662520942122

20 Dieckmann NF, Mauro R, Slovic P. The effects of presenting imprecise probabilities in intelligence forecasts. *Risk Anal* 2010;**30**:987–1001. doi:10.1111/j.1539-6924.2010.01384.x

21 Jenkins SC, Harris AJL, Lark RM. When unlikely outcomes occur: the role of communication format in maintaining communicator credibility. *J Risk Res* 2019;**22**:537–54. doi:10.1080/13669877.2018.1440415

22 Batteux E, Bilovich A, Johnson SGB, *et al.* When precision backfires: The effects of unwarranted precision on consumer loyalty. *Work Pap* 2020.

23 Lazarus J V, Ratzan S, Palayew A, *et al.* Hesitant or not? A global survey of potential acceptance of a COVID-19 vaccine Jeffrey. *MedRxiv* 2020;**preprint**:1–31. doi:10.1038/s41591-020-1124-9

24 [dataset] Batteux E. Communicating medical uncertainty. Open Sci. Framew. 2020.https://osf.io/c73px/

For peer review only

For peer review only - http://bmjopen.bmj.com/site/about/guidelines.xhtml

25 Tuckett D, Nikolic M. The role of conviction and narrative in decision-making under radical uncertainty. *Theory Psychol* 2017;**27**:501–23. doi:10.1177/0959354317713158

26 Wright L, Steptoe A, Fancourt D. What predicts adherence to COVID-19 government guidelines ? Longitudinal analyses of 51 , 000 UK adults . *MedRxiv* 2020.

27 Martin LR, Petrie KJ. Understanding the Dimensions of Anti-Vaccination Attitudes: the Vaccination Attitudes Examination (VAX) Scale. *Ann Behav Med* Published Online First: 2017. doi:10.1007/s12160-017-9888-y

28 Hayes AF. PROCESS: A versatile computational tool for observed variable mediation, moderation, and conditional process modeling. White Pap. 2012. doi:978-1-60918-230-4

29 Paul E, Steptoe A, Fancourt D. Attitudes towards vaccines and intention to vaccinate against COVID-19: Implications for public health communications. *Lancet Reg Heal - Eur* 2020;**1**. doi:10.1016/j.lanepe.2020.100012

30 Peiris M, Leung GM. What can we expect from first-generation COVID-19 vaccines? *Lancet* 2020;**6736**:20–1. doi:10.1016/s0140-6736(20)31976-0

31 Al-Hasan A, Khuntia J, Yim D. Does Seeing What Others Do Through Social Media Influence Vaccine Uptake and Help in the Herd Immunity Through Vaccination? A Cross-Sectional Analysis. *Front Public Heal* 2021;**9**:1–13. doi:10.3389/fpubh.2021.715931

32 Edelman. Edelman Trust Barometer 2020. Published Online First: 2020. doi:https://www.edelman.com/trustbarometer

33 Tuckett D. Explanatory models and conviction narratives. In: Christmas S, Michie S, West R, eds. *Thinking about Behaviour Change: An Interdisciplinary Dialogue*. Silverback Publishing 2015.

34 Tuckett D, Boulton M, Olson C, *et al. Meetings between experts: An approach to sharing ideas in medical consultations.* London and New York: : Tavistock Publications 1985.

35 Blastland M, Freeman ALJ, Van Der Linden S, *et al.* Five rules for evidence communication. *Nature* 2020;**587**. doi:10.1038/d41586-020-03189-1

36 Sunstein C, Altieri E, Kim M, *et al.* Behavioural considerations for acceptance and uptake of COVID-19 vaccines. 2020.

For peer review only

Figure legends

Figure 1: CONSORT flow diagram depicting the phases of participant recruitment and analysis.

Figure 2: Vaccination intention and perceived vaccine effectiveness before receiving conflicting information (i.e. after the vaccine announcement) and after receiving conflicting information by announcement certainty.

Figure 3: Trust and confidence in the government representative who made the vaccine announcement before receiving conflicting information (i.e. after the vaccine announcement) and after receiving conflicting information by announcement certainty.

Figure 4: Relationship between announcement certainty and vaccination intention after receiving conflicting information mediated by trust in government representative and perceived vaccine effectiveness. * refers to $p<.05$, ** refers to $p<.01$, *** refers to $p<.001$.

Figure 5: Emotions before receiving conflicting information (i.e. after the vaccine announcement) and after receiving conflicting information by announcement certainty.

For peer review only

For peer review only - http://bmjopen.bmj.com/site/about/guidelines.xhtml

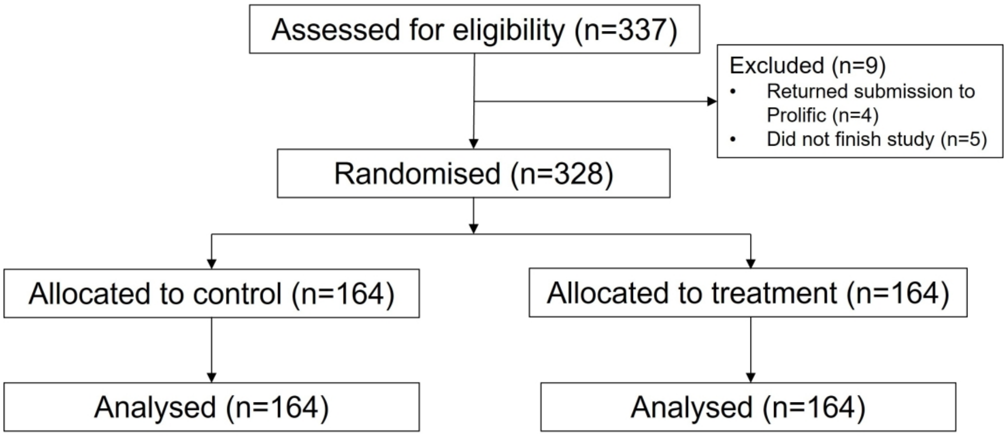

Figure 1: CONSORT flow diagram depicting the phases of participant recruitment and analysis.

212x93mm (330 x 330 DPI)

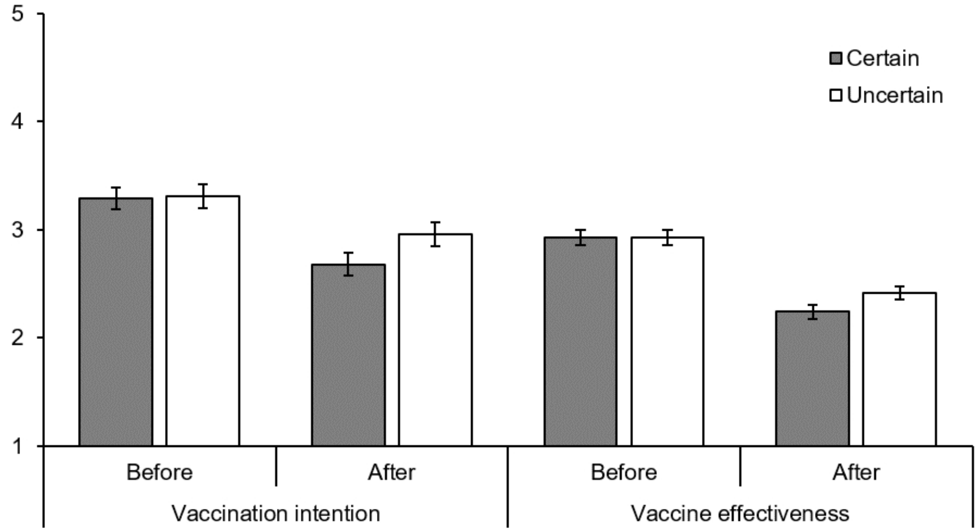

Figure 2: Vaccination intention and perceived vaccine effectiveness before receiving conflicting information (i.e. after the vaccine announcement) and after receiving conflicting information by announcement certainty.

396x218mm (59 x 59 DPI)

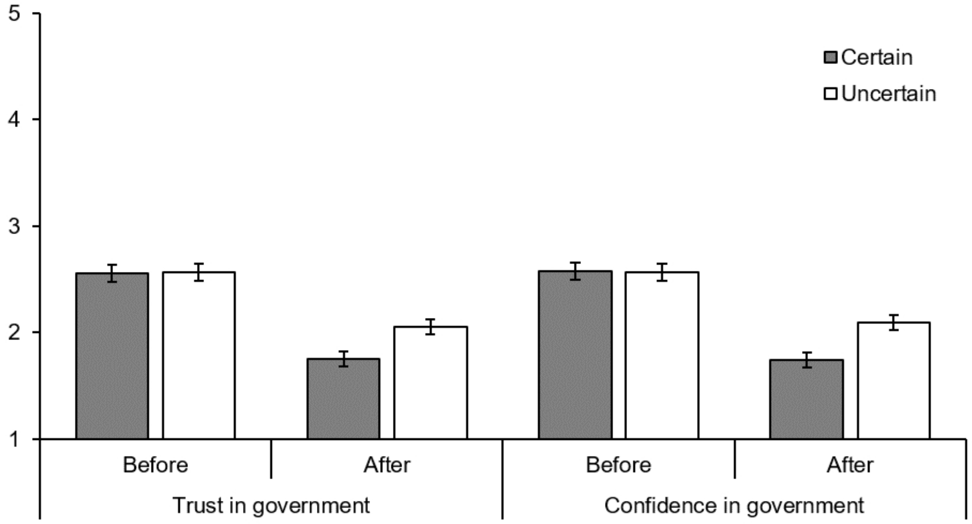

Figure 3: Trust and confidence in the government representative who made the vaccine announcement before receiving conflicting information (i.e. after the vaccine announcement) and after receiving conflicting information by announcement certainty.

401x218mm (59 x 59 DPI)

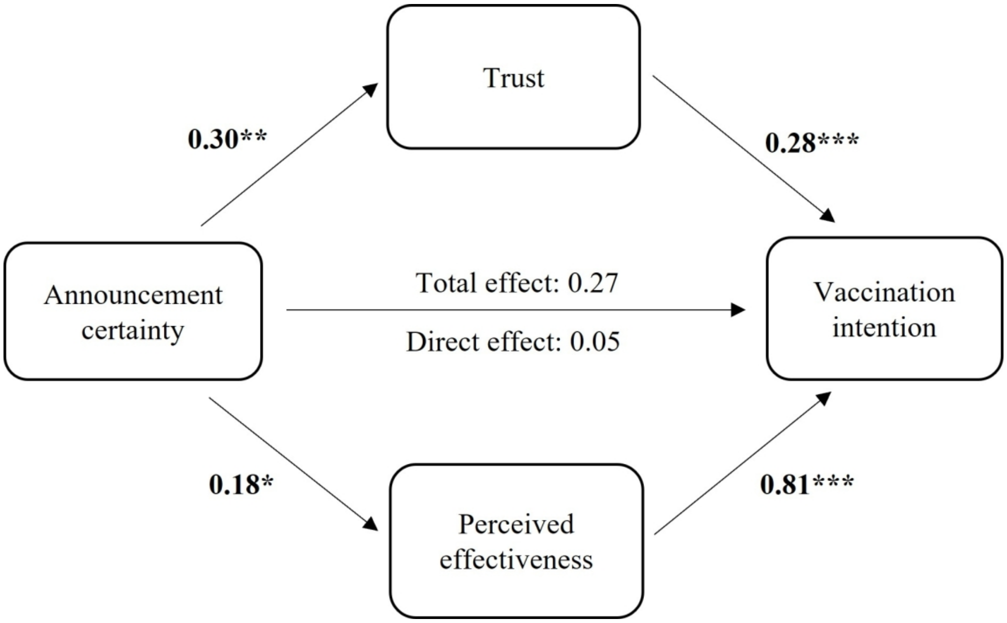

Figure 4: Relationship between announcement certainty and vaccination intention after receiving conflicting information mediated by trust in government representative and perceived vaccine effectiveness. * refers to p<.05, ** refers to p<.01, *** refers to p<.001.

144x88mm (330 x 330 DPI)

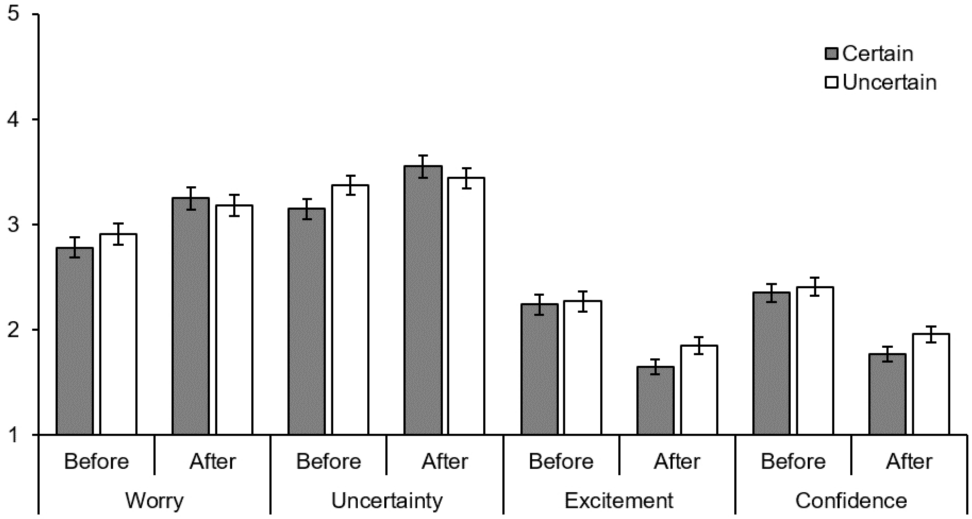

Figure 5: Emotions before receiving conflicting information (i.e. after the vaccine announcement) and after receiving conflicting information by announcement certainty.

404x218mm (59 x 59 DPI)

Appendix: Further analyses on outcome variables

We investigated whether announcement certainty, demographics (age, gender, education) and COVID-19 related beliefs (trust in government, perceived certainty of COVID-19 science, vaccination beliefs) affected our outcome variables (vaccination intention, perceived vaccine effectiveness, trust and confidence in the government official, worry, uncertainty, excitement and confidence). We conducted multiple linear regressions on the differences in each outcome variable between ratings before and after receiving conflicting information (see Table A1). As in our analyses in the main paper, those who received the certain announcement reported the greatest differences between before and after receiving conflicting information. Beliefs towards vaccination also had an effect across most variables, whereby those with more positive beliefs towards vaccination experienced greater differences between before and after receiving conflicting information. This suggests that people who have more positive vaccination beliefs are more likely to be disappointed after receiving conflicting information about vaccine effectiveness. Perhaps this is due to them having greater expectations of vaccine effectiveness and being more surprised once those expectations are not fulfilled.

Table A1: Effects of certainty, demographics and COVID-19 beliefs on differences in outcome before and after conflicting information

	Vaccine		Government		Emotions			
	Vaccination Intention	Effectiveness	Trust	Confidence	Worry	Uncertainty	Excitement	Confidence
Announcement certainty	*B*=-0.27 (0.08)**	*B*=-0.18 (0.08)*	*B*=-0.32 (0.09)***	*B*=-0.39 (0.10)***	*B*=0.23 (0.10)*	*B*=0.35 (0.11)**	*B*=-0.20 (0.09)*	*B*=-0.16 (0.09)
Age	*B*<0.01 (<0.01)	*B*<0.01 (<0.01)	*B*<0.01 (<0.01)	*B*=0.01 (<0.01)	*B*<0.01 (<0.01)	*B*<0.01 (<0.01)	*B*<0.01 (<0.01)	*B*=0.01 (<0.01)**
Gender	*B*=0.08 (0.09)	*B*=0.17 (0.08)*	*B*=0.22 (0.10)*	*B*=0.07 (0.10)	*B*=0.11 (0.10)	*B*=0.03 (0.12)	*B*=0.12 (0.10)	*B*=0.06 (0.10)
Education	*B*<0.01 (0.05)	*B*=-0.03 (0.04)	*B*=-0.05 (0.05)	*B*=0.01 (0.05)	*B*=-0.02 (0.05)	*B*=-0.10 (0.06)	*B*=-0.02 (0.05)	*B*=0.04 (0.05)
Trust in government	*B*=0.08 (0.05)	*B*=0.04 (0.04)	*B*=0.09 (0.05)	*B*=0.14 (0.05)**	*B*<0.01 (0.05)	*B*=-0.03 (0.06)	*B*=0.04 (0.05)	*B*=0.04 (0.05)
Science certainty	*B*=-0.01 (0.01)	*B*<0.01 (0.01)	*B*=0.01 (0.01)	*B*=-0.01 (0.01)	*B*=-0.01 (0.01)	*B*=-0.01 (0.02)	*B*<0.01 (0.01)	*B*=-0.01 (0.01)

Vaccine beliefs	*B*=0.01 (0.01)*	*B*=0.01 (0.01)*	*B*=0.02 (0.01)***	*B*=0.03 (0.01)***	*B*<0.01 (<0.01)	*B*<0.01 (<0.01)	*B*=0.02 (0.01)***	*B*=0.02 (0.01)***
Model	**F=3.01, R^2=6.2%****	**F=2.67, R^2=5.5%***	**F=7.46, R^2=14%*****	**F=8.66, R^2=15.9%*****	F=0.99, R^2=2.1%	F=1.80, R^2=3.8%	**F=4.36, R^2=8.7%*****	**F=5.69, R^2=9.1%*****

Note: Each outcome variable represents the difference in rating before and after receiving conflicting information. Predictor variables are announcement certainty (1=certain, 2=uncertain), age, gender (1=male, 2=female, 3=non-binary), education (1=GCSE or equivalent, 2=A-level or equivalent, 3=undergraduate degree, 4=postgraduate degree), trust in the UK government (scores range from 1-5), beliefs about the certainty of COVID-19 related science (scores range from 3-21), positive beliefs towards vaccination (scores range from 12-60). * refers to $p<.05$, ** $p<.01$, *** $p<.001$.

For peer review only

CONSORT 2010 checklist of information to include when reporting a randomised trial*

Section/Topic	Item No	Checklist item	Reported on page No
**Title and abstract**			
	1a	Identification as a randomised trial in the title	1
	1b	Structured summary of trial design, methods, results, and conclusions (for specific guidance see CONSORT for abstracts)	2
**Introduction**			
Background and objectives	2a	Scientific background and explanation of rationale	1-4
	2b	Specific objectives or hypotheses	4-5
**Methods**			
Trial design	3a	Description of trial design (such as parallel, factorial) including allocation ratio	7
	3b	Important changes to methods after trial commencement (such as eligibility criteria), with reasons	NA
Participants	4a	Eligibility criteria for participants	7
	4b	Settings and locations where the data were collected	7
Interventions	5	The interventions for each group with sufficient details to allow replication, including how and when they were actually administered	9
Outcomes	6a	Completely defined pre-specified primary and secondary outcome measures, including how and when they were assessed	9
	6b	Any changes to trial outcomes after the trial commenced, with reasons	NA
Sample size	7a	How sample size was determined	7-8
	7b	When applicable, explanation of any interim analyses and stopping guidelines	NA
Randomisation:			
Sequence generation	8a	Method used to generate the random allocation sequence	7
	8b	Type of randomisation; details of any restriction (such as blocking and block size)	7
Allocation concealment mechanism	9	Mechanism used to implement the random allocation sequence (such as sequentially numbered containers), describing any steps taken to conceal the sequence until interventions were assigned	7
Implementation	10	Who generated the random allocation sequence, who enrolled participants, and who assigned participants to interventions	7
Blinding	11a	If done, who was blinded after assignment to interventions (for example, participants, care providers, those	7

For peer review only

		assessing outcomes) and how	
	11b	If relevant, description of the similarity of interventions	NA
Statistical methods	12a	Statistical methods used to compare groups for primary and secondary outcomes	9-14
	12b	Methods for additional analyses, such as subgroup analyses and adjusted analyses	9-14
**Results**			
Participant flow (a diagram is strongly recommended)	13a	For each group, the numbers of participants who were randomly assigned, received intended treatment, and were analysed for the primary outcome	7
	13b	For each group, losses and exclusions after randomisation, together with reasons	NA
Recruitment	14a	Dates defining the periods of recruitment and follow-up	7
	14b	Why the trial ended or was stopped	7
Baseline data	15	A table showing baseline demographic and clinical characteristics for each group	8
Numbers analysed	16	For each group, number of participants (denominator) included in each analysis and whether the analysis was by original assigned groups	9
Outcomes and estimation	17a	For each primary and secondary outcome, results for each group, and the estimated effect size and its precision (such as 95% confidence interval)	9-14
	17b	For binary outcomes, presentation of both absolute and relative effect sizes is recommended	NA
Ancillary analyses	18	Results of any other analyses performed, including subgroup analyses and adjusted analyses, distinguishing pre-specified from exploratory	12
Harms	19	All important harms or unintended effects in each group (for specific guidance see CONSORT for harms)	NA
**Discussion**			
Limitations	20	Trial limitations, addressing sources of potential bias, imprecision, and, if relevant, multiplicity of analyses	15-16
Generalisability	21	Generalisability (external validity, applicability) of the trial findings	15-16
Interpretation	22	Interpretation consistent with results, balancing benefits and harms, and considering other relevant evidence	15
**Other information**			
Registration	23	Registration number and name of trial registry	6
Protocol	24	Where the full trial protocol can be accessed, if available	6
Funding	25	Sources of funding and other support (such as supply of drugs), role of funders	18

*We strongly recommend reading this statement in conjunction with the CONSORT 2010 Explanation and Elaboration for important clarifications on all the items. If relevant, we also recommend reading CONSORT extensions for cluster randomised trials, non-inferiority and equivalence trials, non-pharmacological treatments, herbal interventions, and pragmatic trials. Additional extensions are forthcoming: for those and for up to date references relevant to this checklist, see www.consort-statement.org.

For peer review only - http://bmjopen.bmj.com/site/about/guidelines.xhtml

For peer review only
